# Quantitative fluorescence loss in photobleaching for analysis of protein transport and aggregation

**DOI:** 10.1186/1471-2105-13-296

**Published:** 2012-11-13

**Authors:** Daniel Wüstner, Lukasz M Solanko, Frederik W Lund, Daniel Sage, Hans J Schroll, Michael A Lomholt

**Affiliations:** 1 Department of Biochemistry and Molecular Biology, University of Southern Denmark, Campusvej 55, Odense M, DK-5230, Denmark; 2 Biomedical Imaging Group, Ecole Polytechnique Fédérale de Lausanne (EPFL), Lausanne, CH-1015, Switzerland; 3 Institute for Mathematics and Computer Science (IMADA), University of Southern Denmark, Odense M, DK-5230, Denmark; 4 Department of Physics, Chemistry and Pharmacy, MEMPHYS Center for Biomembrane Physics, University of Southern Denmark, Odense M, DK-5230, Denmark

**Keywords:** Mathematical model, Crowding, Protein aggregation, Fractal kinetics, Rate coefficient, Multi-compartment, Neurodegeneration

## Abstract

**Background:**

Fluorescence loss in photobleaching (FLIP) is a widely used imaging technique, which provides information about protein dynamics in various cellular regions. In FLIP, a small cellular region is repeatedly illuminated by an intense laser pulse, while images are taken with reduced laser power with a time lag between the bleaches. Despite its popularity, tools are lacking for quantitative analysis of FLIP experiments. Typically, the user defines regions of interest (ROIs) for further analysis which is subjective and does not allow for comparing different cells and experimental settings.

**Results:**

We present two complementary methods to detect and quantify protein transport and aggregation in living cells from FLIP image series. In the first approach, a stretched exponential (StrExp) function is fitted to fluorescence loss (FL) inside and outside the bleached region. We show by reaction–diffusion simulations, that the StrExp function can describe both, binding/barrier–limited and diffusion-limited FL kinetics. By pixel-wise regression of that function to FL kinetics of enhanced green fluorescent protein (eGFP), we determined in a user-unbiased manner from which cellular regions eGFP can be replenished in the bleached area. Spatial variation in the parameters calculated from the StrExp function allow for detecting diffusion barriers for eGFP in the nucleus and cytoplasm of living cells. Polyglutamine (polyQ) disease proteins like mutant huntingtin (mtHtt) can form large aggregates called inclusion bodies (IB’s). The second method combines single particle tracking with multi-compartment modelling of FL kinetics in moving IB’s to determine exchange rates of eGFP-tagged mtHtt protein (eGFP-mtHtt) between aggregates and the cytoplasm. This method is self-calibrating since it relates the FL inside and outside the bleached regions. It makes it therefore possible to compare release kinetics of eGFP-mtHtt between different cells and experiments.

**Conclusions:**

We present two complementary methods for quantitative analysis of FLIP experiments in living cells. They provide spatial maps of exchange dynamics and absolute binding parameters of fluorescent molecules to moving intracellular entities, respectively. Our methods should be of great value for quantitative studies of intracellular transport.

## Background

Quantitative fluorescence microscopy witnesses an increasing demand for computational methods allowing for interpretation of the complex data generated by this imaging technique. To determine intracellular transport dynamics of proteins and lipids tagged with suitable fluorophores, one often relies on perturbing the steady state distribution of the probe and following the dynamics of re-establishing a steady state. One example for this approach are pulse-chase experiments, where a fluorescent ligand, like a dye-tagged transferrin (Tf) or fluorescent lipoprotein binds to its receptor at the cell surface at the start of the experiment (*t* = 0), and the transport of the fluorescent-ligand-receptor complex to the target organelle during the course of endocytosis is followed over time by quantitative fluorescence microscopy [1-3]. Pre-labeling of the target organelle with a suitable marker with different spectral characteristics allows, together with appropriate image analysis tools, to measure transport kinetics of the molecule of interest by multicolour fluorescence microscopy. Compartment modelling based on ordinary differential equations can be used to determine inter-organelle transport kinetics. For example, trafficking kinetics of fluorescent Tf and lipoproteins to sorting endosomes and the endocytic recycling compartment (ERC) have been measured in this way in various cell types [1-8]. Similarly, using a temperature-sensitive folding mutant, export of a viral protein tagged with eGFP has been analyzed by this approach [9].

An alternative way to perturb the steady state does not rely on pulse-labeling molecules in a particular compartment and is often used to elucidate transport dynamics along the secretory pathway or between nucleus and cytoplasm [10-16]. Selective photodestruction of fluorescence of the molecule of interest in one organelle and measuring fluorescence recovery after photobleaching (FRAP) of unbleached molecules into the area reveals transport kinetics and provides information about a possible immobile fraction of the labeled molecules in the target organelle [17]. A variant of FRAP is continuous photobleaching (CP) introduced by Peters et al. in 1981 [18]. In this approach, a small area of the cell is continuously illuminated, and the fluorescence decay in this region due to photobleaching and transport is monitored. Similar as in FRAP, diffusion constants, and with extensive modelling efforts, binding parameters or flow components can be estimated from CP measurements [18-20]. Both techniques, however, infer the transport parameters solely from fitting a time-dependent function to experimental data. Thus, any locally varying transport properties in the cell cannot be directly monitored, since FRAP and CP curves do not depend explicitly on spatial coordinates. Consequently, one often finds model redundancy; i.e., that various mathematical models can describe a measured FRAP curve equally well [21]. Another complication in both, FRAP and CP is that the user has only very limited impact on the time hierarchy of the experiment. While varying the size of the bleach area allows for controlling the diffusion time, if binding and diffusion take place on different time scales, the user cannot isolate one of the processes by adjusting the time resolution of the measurement.

A method related to FRAP is fluorescence loss in photobleaching (FLIP). In FLIP, a region is repeatedly illuminated by an intense laser pulse, while images are taken with reduced laser power between the bleaches. A pause between the laser pulses allows for some recovery in the bleached region. The duration of the pause can be set by the user, who thereby can control the time resolution of the experiment. Repeating this protocol several times creates a sink for the fluorescent molecules in the local environment being in continuous exchange with the bleached region [17]. A decrease of fluorescence of dye-tagged molecules outside the bleached area allows for assessing continuity between intracellular compartments, and in principal, for measuring the kinetics of recruitment to the bleached region from various cellular areas. Accordingly, FLIP has the potential to include spatial information in the computational analysis. However, only very few FLIP analysis efforts published so far try to include the whole image information into analysis of FLIP image sets: In one study, van Gemert et al. (2009) used independent component analysis to decompose FLIP image series into static and dynamic components [22]. Very recently, van de Giessen et al. (2012) used a monoexponential decay model on a pixel basis combined with image registration and denoising to measure FL in FLIP image series [23]. The widely used standard approach for analysis of FLIP data is to define rectangular regions of interest (ROI) at different sites in the cell and to quantify the mean fluorescence in these ROI as function of time [17]. The user chooses the number, location and size of the ROI’s based on visual inspection of the image sets. This generates a very subjective element in the data analysis.

We present two new approaches to quantify FLIP experiments reliably; either on an image basis to detect areas of different probe mobility, or by physical modelling of FL from moving entities. In the first section, we demonstrate that a stretched exponential (StrExp) function can be fitted to the decaying intensity at each pixel position in the bleached and non-bleached region of the cell. This is used to detect diffusion-limited depletion zones around the bleached area in cells expressing enhanced green fluorescent protein (eGFP). Pixel-wise fitting of the StrExp function to FLIP image sets also allows us to determine local heterogeneity in nucleocytoplasmic transport of eGFP. In the second section, we measure FL kinetics in moving inclusion bodies (IB’s) by combining FLIP with single-particle tracking. From that data, we infer exchange parameters of mutant Huntingtin tagged with eGFP (eGFP-mtHtt) by multi-compartment (MC) modelling of binding/release and fluorescence attenuation due to photobleaching. The presented methods should find wide application in quantitative cell biology and especially in analysis of protein aggregation in neurodegenerative diseases.

## Results

### The stretched exponential function as empirical decay law for transport studies

The StrExp function is an empirical decay function with broad applications in modelling of physical, photochemical and biophysical data [24-28]. It can be considered as a generalization of the exponential function, since the stretching parameter *h* gives *a* time-dependent rate constant, called a rate coefficient (see Equation 1 in Methods and Additional file 1: Figure S1B and Equation S4) [27]. Rudolf Kohlrausch first introduced this function in 1854 to describe the discharge of a capacitator, which is why it is sometimes also called the Kohlrausch function [29]. It has been demonstrated that many physical relaxation processes can be described by a StrExp function including frequency-dependent dielectric constants of polymeric systems and glasses [30,31], luminescence decays of macromolecules in heterogeneous environments [27,28], luminescence quenching and resonance energy transfer in disordered media [24,27], spin-relaxation [24] and mechanical stress relaxation in crystals [32]. All of these processes have in common that a physical system is perturbed for a short time followed by relaxation towards a new equilibrium or steady state value, respectively. In some cases, the StrExp function can be used to discern a physical mechanism underlying the observed relaxation process [24]. More often, it is used as empirical fitting function to describe an experiment in quantitative terms [26-28,32]. Here, we use the StrExp function as empirical function to describe FLIP image sets on a pixel basis. In a classical FLIP experiment, the perturbation is caused by a series of intense bleach pulses in the selected ROI. We will consider two limiting cases of a FLIP experiment and demonstrate that the StrExp function concurs with simulated time courses for both situations.

### Analysis of diffusion-limited FLIP experiments using the StrExp function

**Figure 1:**
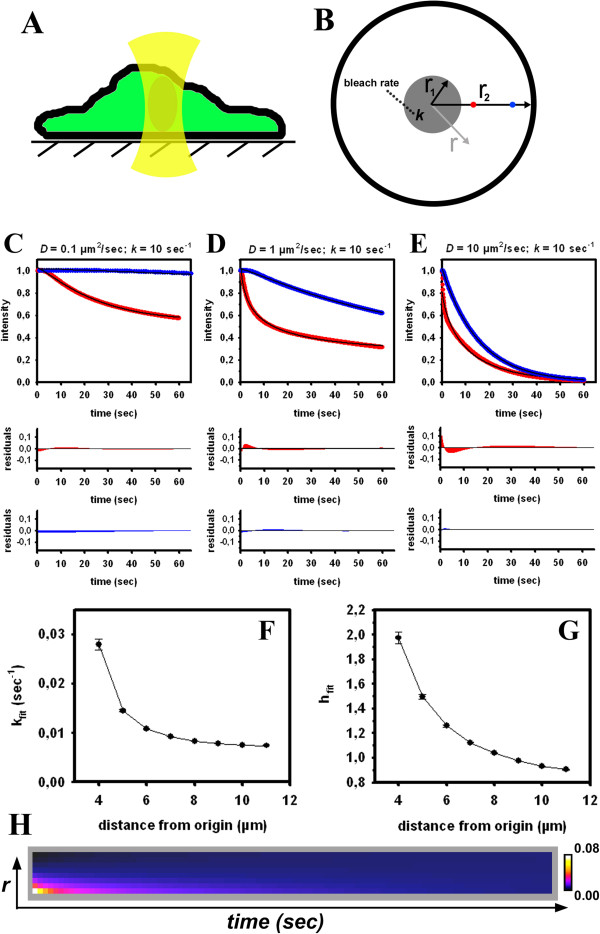
**Simulation of homogenous diffusion and fitting with the StrExp function.A**, Sketch of the FLIP experiment with the cell attached to a surface and filled with eGFP (green) and the cylindrical laser beam focused in the cell center (yellow). **B**, Geometry of the analytical model for the reaction diffusion system in Eq. 6 to model the FLIP experiment. The cell is assumed to be a flat cylinder with a radius, r_2_ = 12 μm. The central bleached region with radius r_1_ = 3 μm is also cylindrical covering the whole cell height (grey shaded area). **C-E**, The model was solved analytically and simulated for two positions outside the bleached area at a distance of 5 μm (red dot in B and red symbols in **C-E**) and 10 μm (blue dot in B and blue symbols in **C-E**) from the origin, respectively. Simulations were performed with a rate constant for the intended bleaching process of *k* = 10 sec^-1^ and diffusion constants of D = 0.1 μm^2^/sec (**C**), D = 1 μm^2^/sec (**D**) and D = 10 μm^2^/sec (**E**). A non-linear regression with the StrExp function (black lines) was performed in SigmaPlot (upper panels) including the residuals of the fit (lower panels). **F**, **G**, time courses were simulated for D = 0.1 μm^2^/sec as a function of distance from the origin and fitted to the StrExp function. Fitted parameters including standard deviation of the fit are plotted for the rate constant (‘*k*_fit_’; F) and stretching parameter (‘*h*_fit_’; G). **H**, rate coefficients calculated according to Eq. S4 for the parameters in panels **F**, **G** as function of distance from bleach ROI (starting at 4 μm from origin and indicated as ‘***r***’ on the ordinate in H) over time. The scale bar shows rate coefficients color-coded using a FIRE-LUT

When the pause between individual bleaches is short compared to the molecular transport rates, the measured FL becomes limited by diffusion of the molecules towards the bleached area. Developing a physical model for this situation requires taking the exact location of the bleach ROI into account. Assuming that binding/dissociation events are very fast compared to molecular diffusion the process is governed by Equation 6 which can be solved (see Methods and Appendix 1). The diffusion model assumes cylindrical cell geometry and cylindrical bleach in the centre of the cell within 3 μm around the origin performed over the whole cell height (Figure 1A). Under these assumptions, we have a 2D-problem, which is reasonable for bleaching experiments in confocal microscopy due to the large depth of field of the focussed laser beam [33]. The radial symmetry of the diffusion problem implies that the FL is only a function of the magnitude of the radius vector $\vec{r}$ but not of its direction. Thus, it is sufficient to analyze some points with varying distance from the centre. Note that this model is an oversimplification of a real FLIP experiment (i.e., cylindrical versus conical bleach profile, radial symmetric cell etc.), with the sole intention to assess the ability of StrExp function for describing diffusion-limited bleaching processes. We have experimentally verified that the bleach profile of the laser is in fact only a cylinder, when using an objective with low numerical aperture (i.e., 40x with NA=0.7). For the high resolution objectives used here (i.e., 40x or 63x with NA=1.2), the bleach profile gives a double cone, as verified using fixed cells expressing eGFP (see Additional file 1: Figure S2). We found in additional FLIP experiments with cells expressing eGFP that the bleach profile has no impact on the ability of the StrExp function to fit the data. The lower NA objective, however, gave much worse signal-to-noise ratio for the same scanning intensity, which deteriorates the fitting performance (not shown). Additional simulations using a 3D geometry showed that the StrExp can fit synthetic FLIP image sets with either a conical or a cylindrical bleach profile equally well (see Additional file 1: Figure S3). Fast bleaching causes the generation of a depletion zone surrounding the bleached area (i.e., for *r*_1_ < *r* < *r*_2_ in Figure 1B). The depletion zone is characterized by faster FL for positions close to the bleached region than for positions further away. This is illustrated in Figure 1 for two selected points located outside the bleached region of the cell; i.e., 5 μm (red dot and red curves) and 10 μm (blue dot and blue curves). The depletion zone is more pronounced, the slower the diffusion coefficient is compared to the bleach rate (Figure 1C-E). We used the StrExp function to empirically fit the simulated FL at 5 and 10 μm radial distances (Figure 1C-E). The StrExp fits diffusion-limited FL in both positions well, as inferred by low residuals between data and regression. The regression result becomes better the larger the distances of the analyzed point from the bleached area (i.e., at 10 μm). Closer to the bleached area, i.e. at 5 μm, the StrExp function cannot account for the almost biphasic decay (see large residuals up to 10 sec). Moreover, close to the bleached region the fit is better for smaller diffusion constants (compare residuals in red in Figure 1C-E).

Diffusion is a process in space and time, while the StrExp function provides kinetic parameters only at one location (i.e., it does not depend on position; we just map the function over all pixel positions $\vec{r}$ = (*x*, *y*), see Equation 1). Thus, we need to make sure that there is a monotonic relationship between the parameters of the fit function at various positions and the output of the diffusion model for a given parameter combination. We found that both, the rate constant and the stretching parameter of the StrExp function decline monotonically for increasing distance, *r*, from the center of the cell (Figure 1F, G). Moreover, for increasing distances from the cell centre, the StrExp function switches from a stretched (*h* > 1) to a compressed exponential (*h* < 1) (Figure 1G). The bleaching process in these FLIP simulations can be considered as diffusion-limited chemical reaction destroying fluorophores selectively in the bleached ROI. The number of fluorophores, n(t), drops due to localized bleaching, for which one can set *dn*(*t*)/*dt* = − *k*(*t*) · *n*(*t*). The time-dependent rate coefficient, *k*(*t*), models the diffusion-limited destruction of fluorophores [34-36]. In the StrExp function, we have $k\left(t\right)=\frac{1}{h\cdot \tau_{0}}\cdot {\left(\frac{t}{\tau_{0}}\right)}^{\frac{1}{h}-1}$ with *h* > 1 in the bleached ROI and the depletion zone and h < 1 at longer distances from the bleach spot (see Additional file 1: Equations. S1-S4). Accordingly, the rate coefficient describing the kinetics of FL decreases for the stretched case (in the bleached ROI and the depletion zone) and increases for the compressed case of the fitting function (distant from the bleach spot). This is illustrated in Figure 1H, where the rate coefficients were calculated using the fitting parameters of the StrExp function (compare Figure 1F, G). In and close to the bleach spot, the bleaching reaction slows down over time, since the fluorescent molecules being located further apart must travel longer distances to reach the bleach ROI. This is equivalent to a decreasing rate coefficient and *h* > 1, respectively. Vice versa, for regions more distant from the bleached ROI, there is a delay before FL takes place, which is characterized by compressed exponentials (h < 1) and an increasing rate coefficient. If bleaching is slow compared to the probe diffusion, the intensity decay at each position outside the bleached ROI is solely governed by the bleach rate constant, and we recover a mono-exponential decay at each position (i.e., bleach-limited FLIP, not shown). Table 1 summarizes the possible scenarios in diffusion-limited FLIP experiments and the possible outcome of the StrExp fit to the data.

**Table 1:** Relationships between parameter combinations of the StrExp function fitted to experimental FLIP data and the time hierarchy of photobleaching and possible intracellular transport processes

**Scenario**	**Parameters recovered from the fit of the StrExp function to experimental fluorescence loss in FLIP image sets**			
	**Stretching (*h*)**	**Time constant (*τ*)**	**Rate coefficient (*k*(t))**	**Explanation**
***Diffusion-limited FL***	Diffusion of molecules to the bleached ROI is slower than the bleaching process. This results in a ’depletion zone’ around the bleached area. Binding and release, if any, are faster than diffusion.			
**Inside ROI**	1 < *h* < 2	Dependent on bleach rate constant and diffusion constant*.	Decreasing over time.	Fluorophores get depleted by the bleaching acting as diffusion-limited reaction.
**Outside ROI**	~0.5 < *h* < 1	Dependent on diffusion constant and bleach rate constant*.	Increasing over time.	Compressed decay, because diffusion to the bleach ROI causes delayed response.
***Bleach-limited FL***	Diffusion of molecules to the bleached ROI as well as eventual binding and release are faster than the bleaching process. The only process causing FL is the repeated bleaching inside the ROI.			
**Inside ROI**	~ 1	Dependent on bleach rate constant only.	Constant over time and equal to 1/τ.	Approx. mono-exponential decay determined by the bleach rate.
**Outside ROI**	~ 1	Dependent on bleach rate constant only.	Constant over time and equal to 1/τ.	Approx. mono-exponential decay determined by the bleach rate.
***Binding***^***#***^ ***-limited FL***	Diffusion is fast but molecules are hindered by binding to cellular organelles or by obstacles. Any spatial fluorescence gradient is rapidly equilibrated and binders/barriers become visible.			
**Inside ROI**	~ 1	Dependent on bleach rate constant only.	Constant over time and equal to 1/τ.	Approx. mono-exponential decay determined by the bleach rate.
**Outside ROI**	~0.8 < *h* < 1	Dependent on release rate constant and bleach rate constant*.	Increasing over time.	Compressed decay, because slow release causes delayed response.

^*^In that order as estimated by parameter sensitivity. ^#^ ‘Binding’ is used synonymous for binding-/barrier-limited FLIP.

**Figure 2:**
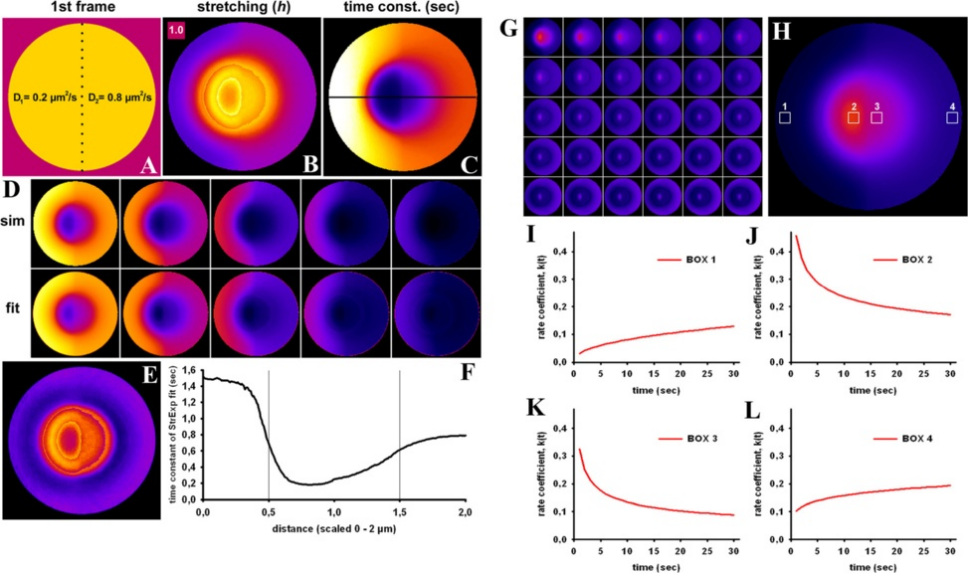
**Simulation of heterogenous diffusion and fitting with the StrExp function.** A 2-dimensional bleaching experiment was simulated on a disk with a circular bleached area of radius *r*_1_=0.5 _μm_ using FeniCS, an automated computational modelling suite (http://www.fenicsproject.org). The bleaching rate is set to *k*=10 sec^-1^ and the diffusion coefficient is *d*_1_=0.2 μm^2^/sec and *d*_2_=0.8 μm^2^/sec on the left and right half disk, respectively (A). Fluorescence loss inside and outside the bleached region was fitted at every pixel position with the StrExp function. For this purpose the PixBleach plugin to ImageJ was used [40]. The regression recovers a map of the stretching parameter, *h*, color-coded between 0.4 to 2.0 (**B**), the time constant distribution color-coded between 1.0 to 15.0 sec (**C**) and the χ^2^-map color-coded between 0 to 50 (**E**). A FIRE-LUT was used for color-coding, where dark blue and yellow indicate lowest and highest values, respectively. The reconstructed stack exactly resembles the simulated data set, as seen in **D** (upper row, simulated data (‘sim‘), lower row, regression (‘fit‘)). **F**, profile of time constants, as estimated from fitting the StrExp function to the simulated FLIP data along the line shown in panel **C**. **G-L**, rate coefficients were calculated on a pixel-by-pixel basis according to $k\left(t\right)=\frac{1}{h\cdot \tau_{0}}\cdot {\left(\frac{t}{\tau_{0}}\right)}^{\frac{1}{h}-1}$ with self-programmed macros to ImageJ. Rate coefficients are color-coded using a FIRE-LUT in the range from 0.017 (dark blue) to 0.97 (yellow). **G**, shows montage of all time points; **H**, first frame of montage with some regions of interest (boxes 1 to 4) used for analysis in panel **I-L**. Rate coefficient as function of time averaged for box 1 (**I**), box 2 (**J**), box 3 (**K**) and box 4 (**L**) from the whole sequence.

To account for heterogeneous intracellular diffusion in FLIP experiments, we performed next a numerical simulation of a circular section of a cell with 2 different diffusion constants (Figure 2 and Additional file 2: Movie S1). On the left half of this 2-μm-sized unit cell, we set the diffusion constant, D, to *D*_1_ = 0.2 μm^2^/sec, while on the right half, we had *D*_2_ = 0.8 μm^2^/sec. Continuity across the boundary ensures that the probe can diffuse across the boundary region. Bleaching occurred within a radius of 0.5 μm around the cell center with a rate constant of *k*_*b*_ = 10 sec^-1^. The simulation was implemented in FEniCS and fitted on a pixel-by-pixel basis to a StrExp function (see Methods). Fluorescence dropped first on the right half of the simulated cell due to fast recruitment of molecules to the bleached area. The empirical fit using the StrExp function accurately recovers the simulated data set, as shown in Figure 2D. Goodness of fit is judged by the χ^2^ parameter, a measure of the weighted sum of the squared errors between data and models. Calculating that parameter on a pixel-by-pixel basis reveals that the fit is less accurate in the bleached ROI (i.e., χ^2^ ~25; Figure 2E). The stretching parameter, *h*, of the StrExp fit was larger than 1 in the bleached ROI and decreased from the depletion zone towards the cell edge (Figure 2B). For the larger diffusion constant, *D*_2_, on the right half of the cell, FL was faster, as indicated by the lower time constants (Figure 2C and F) and higher rate coefficients (Figure 2G-L) compared to the left half of the cell. The slowest and most delayed intensity decay is found on the left cell edge, where *h* ~ 0.65, compared to the right cell border with *h* ~ 0.85. Both values describe compressed exponentials and thereby increasing rate coefficents for the FL with time (compare box 1 and 4 in Figure 2I, L). The spatial gradient of FL kinetics from close to the bleached area towards the cell border is more pronounced for the left half with slow diffusion (Figure 2F). Together, the simulations show that the StrExp function can ‘catch’ all essential elements of diffusion-limited FLIP experiments. From the estimated parameter set, one can recover the complete spatiotemporal map of the FL process. The main characteristics of diffusion-limited FLIP experiments is revealed in the *h*-map of the regression with the StrExp function, i.e., a gradual transition from a stretched decay inside the bleach ROI with *h* > 1 to a compressed decay outside the ROI with *h* < 1. The slower the diffusion is compared to the bleach rate, the larger are the differences of *h*-values along this gradient. Additional simulations for 3D diffusion can be equally well described by the StrExp function (see Additional file 1: Figure S3). 

### Diffusion-limited FLIP of eGFP in the cytoplasm of McA cells

**Figure 3:**
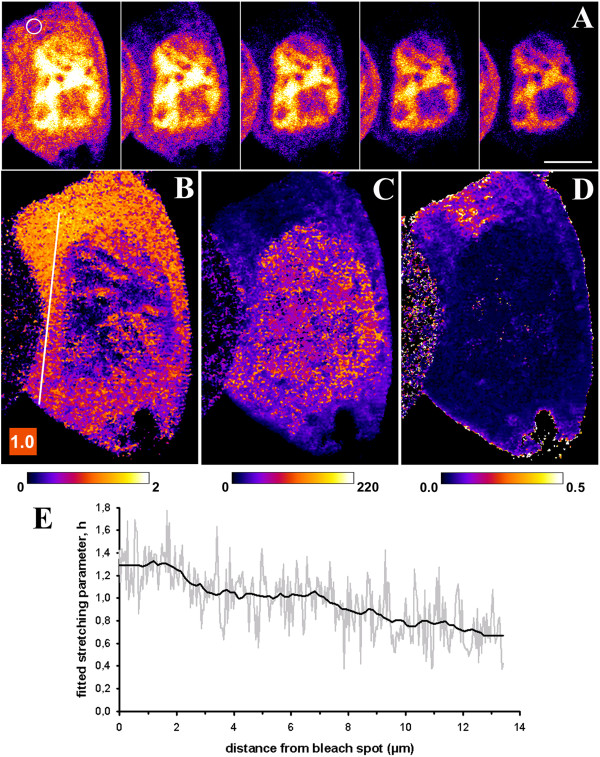
**Diffusion-limited FLIP of eGFP in the cytoplasm of McA cells.** McArdle RH7777 cells expressing eGFP in the cytoplasm and nucleus were placed on a temperature-controlled stage of a confocal microscope maintained at 35 ± 1°C. A 10 pixel diameter circular region in the cytoplasm (white circle) was repeatedly bleached with full laser power, while the whole field was scanned with 0.5% laser output between the bleach scans. This gave a total frame rate of 1.6 sec (see Methods for further details). **A**, montage of the time series with every 20^th^ frame shown. **B-D**, fit of the StrExp function to the data giving a map of the stretching parameter (**B**), the time constant map (**C**) and its reciprocal, the rate constant map (**D**). A FIRE-LUT was used for color-coding, where dark blue and yellow indicate lowest and highest values, respectively. The range of values is given below the images with a color bar without units in B, in sec in **C** and in sec^-1^ in **D**. Bar, 5 μm. **E**, profile of stretching parameters along the line shown in B (grey line, data; black line moving average to smooth the profile).

We aimed for an experimental realization of these predictions in a physiologically relevant setting. Enhanced green fluorescent protein (eGFP) is a small 27 kDa protein which does not bear a nuclear targeting sequence and should not bind to any cellular structures. While its cytoplasmic and nuclear diffusion is very rapid (D ~ 25 μm^2^/sec), eGFP shuttles between nucleus and cytoplasm by passive bidirectional exchange, which is well described by exponential functions [14-16]. We repeatedly bleached fluorescence of eGFP in a small cytoplasmic region (i.e., 5 iterations per bleach at full laser power of 25 mW) and acquired the images at a rate of 1.6 frames/sec, including the bleach. This was fast enough to ensure that cytoplasmic diffusion of eGFP cannot keep up with rapidness of the bleaching process (Figure 3). In compliance with the simulations of Figures 1 and 2 and with Table 1, fitting the StrExp function to this data gives a gradient of stretching parameters, *h*, ranging from *h* ~ 1.7 in the bleached ROI towards *h* ~ 0.7 at the distant side of the cell (Figure 3B and E). This is most obvious in the line profile of the stretching parameter, *h*, drawn as function of distance from the bleach spot (Figure 3E). Thus, FL kinetics of eGFP in this experiment shows a transition from a stretched to a compressed exponential decay when moving away from the bleach spot. The fitted time constants (Figure 3C) and rate constants (Figure 3D) reveal faster decay in the bleached ROI and its immediate surrounding than more distant from it. Together, the parameter maps obtained from fitting the StrExp function to the FLIP data of eGFP reveal sensitively the diffusion-limited regime, even though a depletion zone is not readily apparent in the data. Note, that we had a high detector gain for the experiments shown in Figure 3 to visualize cytoplasmic eGFP with high frame rate and SNR. Fluorescence of nuclear eGFP was therefore saturated in these experiments making exact recovery of rate constants and *h*-values impossible for the nuclear compartment. To further confirm that our method can assess diffusion-limited FLIP data, we performed FLIP experiments using the fluorescent cholesterol probe, BODIPY-cholesterol (BChol) (Additional file 1: Figure S4). BChol diffuses by at least a factor 30 slower than eGFP due to frequent partition into organelle membranes in cells [37]. This allowed us to clearly discern a depletion-zone being accurately described by the StrExp function. Not only FL of BChol in the cytoplasm but also in small moving vesicles could be described by the StrExp suggesting sterol release into the cytoplasm (Additional file 1: Figure S4A-D). Together, the experiments in Figure 3 and Additional file 1: Figure S4 confirm the simulation results: pixel-wise regression of the StrExp function to FLIP image sets not only describes accurately diffusion-limited FLIP experiments but allows also for detecting even faint depletion zones based on the gradient of stretching parameters in the *h*-map.

### Nucleo-cytoplasmic transport of eGFP in McA cells as example of binding/barrier-limited FLIP experiments

In many experimental situations, diffusion is much faster than the photobleaching process in the bleach ROI. Still, transport to the bleached area in FLIP experiments can be hindered by transient binding events or barriers to diffusion, even though the diffusion of a molecule in the aqueous phase of the cytoplasm, i.e., the cytosol, is fast. For example, exchange of proteins between nucleus and cytoplasm has been shown to be limited by transport through the nuclear pore complex but not by diffusion [14-16,38]. Binding-/barrier dominated transport can be modelled by a set of ordinary differential equations, as given in Eqs. 3–5 in Methods. For the given example, the constants *k*_1_ and *k*_−1_ reflect nuclear export and import rate constants, respectively, but the same equations would also describe binding/dissociation-limited FLIP experiments. We confirmed in additional simulations, that the StrExp function is able to describe binding/barrier-dominated FLIP experiments (see Additional file 1: Figure S5, S6, S7 and S8).

**Figure 4:**
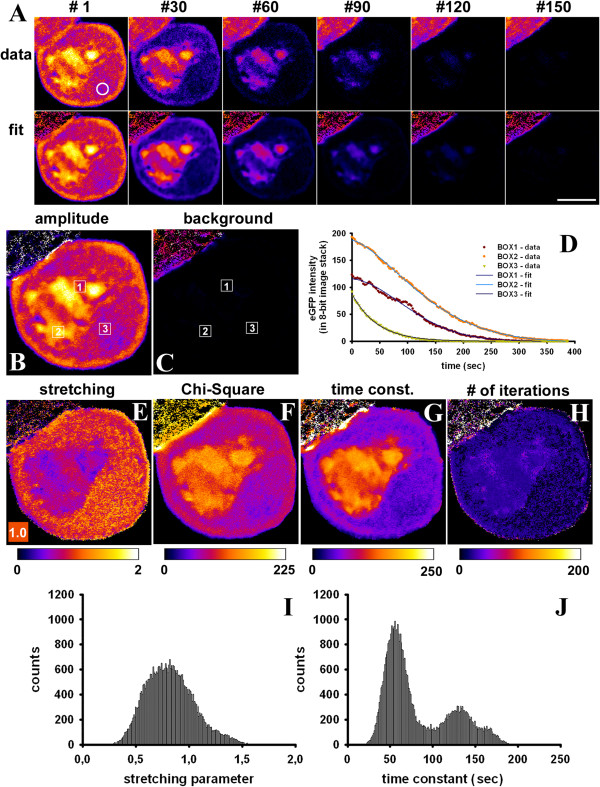
**Barrier-limited FLIP of eGFP shuttling between nucleus and cytoplasm.** McArdle RH7777 cells expressing eGFP in the cytoplasm and nucleus were placed on a temperature-controlled stage of a confocal microscope maintained at 35 ± 1°C. A 30 pixel diameter circular region in the cytoplasm (white circle) was repeatedly bleached with full laser power, while the whole field was scanned with 0.5% laser output between the bleach scans such that the total frame rate was 2.6 sec. A montage of every 30^th^ frame of the data (upper panel, ‘data’) or of the reconstruction from a pixel-wise fit of data to the StrExp function (lower panel, ‘fit’). **B**, amplitude map; **C**, background map, each with three boxes numbered 1 to 3. **D**, FL in these three boxes along the stack (colored symbols) + fit to StrExp function (colored lines). **E-H**, parameter maps produced by PixBleach for: **E**, the stretching parameter (see Eq. 1; the color code for *h* = 1, the mono-exponential case, is given in small box); **F**, χ^2^-values showing the quality of the regression; **G**, time constant map; H, number of iterations. All values are color-coded using a FIRE-LUT, and the range is given below the images with a color bar. The time constant is given in seconds; all other values are without units. **I**, **J**; histograms of the stretching parameters (**I**) and the time constants (**J**) for the cell, shown in **A-H**. Bar, 5 μm.

**Figure 5:**
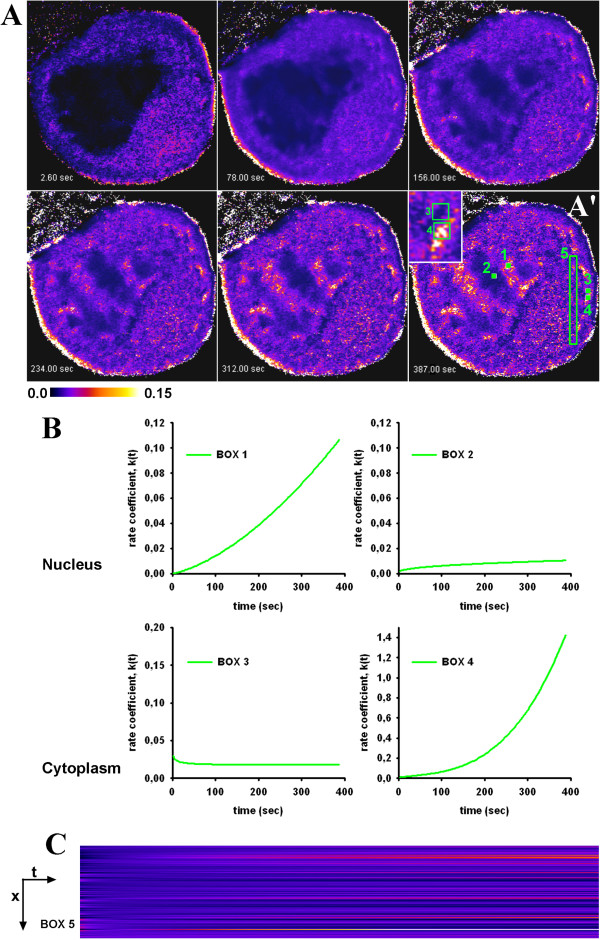
**Pixel-wise rate coefficients of fluorescence loss kinetics of eGFP.** Using the parameter maps of the FLIP analysis for the cell in Figure 4, rate coefficients were calculated for every pixel position, as outlined in Additional file. **A**, the resulting 32-bit image stack was color-coded using a FIRE-LUT, and selected frames of the rate coefficients were plotted (blue and yellow-white indicate low and high rate coefficients, respectively). Areas with accelerating speed of FL turn from blue to yellow-white over time. A’ few regions are highlighted with boxes (numbered ‘1’ to ‘5’) for further analysis. Boxes 1 and 2 were placed in the nucleus, while boxes 3 to 5 were in the cytoplasm (inset shows a zoom for the area containing boxes 3 and 4 close to the right edge of the cell). **B**, rate coefficients as function of time plotted for box 1 to 4. **C**, *x*,*t*-view of the time evolution of the rate coefficients in box 5. See text for further explanations.

For the purpose of an experimental realization of binding/barrier-limited FLIP, we studied nucleo-cytoplasmic shuttling of eGFP by our FLIP approach. A small region in the cytoplasm of McArdle 7777A cells expressing eGFP was repeatedly bleached with a total frame rate including the bleach of 2.6 sec (see white circle in Figure 4A for bleach ROI). Fluorescence loss takes place rather homogeneously in the cytoplasm with no sign for a diffusion-limited depletion zone surrounding the bleach ROI (Figure 4A). Fitting the StrExp function on a pixel-by-pixel basis to that data did not reveal a gradient in the time constant or *h*-map either, supporting that diffusion is not the limiting factor in this FLIP experiment Figure 4E and G). At the edges of the eGFP-filled cytoplasm, the recorded intensity as well as the amplitude, stretching and time constant parameters are slightly larger than in the remaining cytoplasm (Figure 4A-B and E-F). That is likely a consequence of strong differences in refractive index at cell edges, which causes dispersion of the excitation light and thereby an apparent image crisping [39]. It is not caused by the fitting routine, since it was not found in synthetic image sets (compare Figures 1, 2 and Additional file 1: Figure S3, S5, S6, S7 and S8). The FL of eGFP in the nucleus was delayed compared to that in the cytoplasm, likely due to time-limiting transport of the protein through the NPC [14-16]. Fitting the data to a StrExp function gives a very good reconstruction and regression quality (Figure 4A, compare ‘data’ and ‘fit’). We found that all eGFP can be bleached by our FLIP set-up. This is reflected by the absence of background in the non-linear regression of the StrExp model to the data (Figure 4B, C). Some ROI boxes were outlined manually and the experienced FL was fitted to a StrExp function (Figure 4B-D; boxes 1 and 2 in nucleus and box 3 in cytoplasm). Clearly, the StrExp function fits the data very well, with compressed exponential characteristics in the nucleus (box 1: *h* = 0.5427; *k* = 0.0195 frame^-1^; box 2: *h* = 0.6411; *k* = 0.0169 frame^-1^) and an almost mono-exponential decay in the cytoplasm (box 1: *h* = 0.9815; *k* = 0.0571 frame^-1^). Lower stretching with *h* < 1 and large time constants were often found in the nucleus (Figure 4E and G). This is a direct consequence of the rate-limiting transport of eGFP through the nuclear pore complex. Crossing the nuclear membrane causes some delay in the FL in the nucleus compared to regions in the cytoplasm. Accordingly, the rate coefficients calculated from the time constant and *h*-maps increase over time, most prominent in the nucleus (compare Table 1). The difference in time constants between the two cellular compartments is so pronounced that one could even segment cell nuclei and cytoplasm based on the FL time constant of eGFP (see two peaks in time constant histogram, Figure 4J). These results confirm all predictions for simulated barrier-limited FLIP experiments shown in Additional file 1: Figure S5. Notably, intensity in the neighbor cell, which is not affected by the bleaching protocol, remained constant throughout the experiment (Figure 4A). Since our plugin ‘PixBleach’ allows for excluding pixels from the fit which experience an intensity decay lower than some threshold value (‘minimal decay’-option), fluorescence time traces in the neighbor cells were not fitted by the software [40]. By closer inspection, one finds sub-micron granularity in the time constant and *h*-maps for the fits of the StrExp function to the FLIP experiments of eGFP, most pronounced in the nucleus (Figure 4E and G). Calculation of the time evolution of the underlying rate coefficients on a pixel-by-pixel-basis allows for local comparison of decreasing or accelerating FL velocity in adjacent cellular regions (Figure 5 and Additional file 3: Movie 2). For example, nuclear regions with low amounts of eGFP were found to have the most compressed FL kinetics, as indicated by the *h*-map (Figure 4E) and the time evolution of the rate coefficients (Figure 5). The small nuclear area indicated by box 1 in Figure 5A’ (0.33x0.33 μm ~0.11 μm^2^ in size) contains comparable little eGFP, and FL in that region occurred with a time constant of 124.38 sec with strongly accelerating FL speed (*h* = 0.427; Figure 5B). The other nuclear region (box 2, same size) contains more than twice the amount of eGFP and the FL time constant was 178.68 sec. The rate coefficient in box 2 was also increasing over time, but to a much lower extent than in box 1 (*h* = 0.738; Figure 5B). We did the same analysis for two very close areas in the cytoplasm (Figure 5A’ and inset). Fluorescence loss in these regions had similar time constants (i.e. 50.37 sec for box 3 vs. 44.28 sec for box 4; compare Figure 4G), but opposite characteristics of FL (Figure 5B). While in the region highlighted by box 3 FL velocity slowed down over time (*h* = 1.176; Figure 5B), the other area indicated by box 4 was characterized by strongly accelerating speed over time well-described by a compressed exponential function (*h* = 0.666; Figure 5B and see inset in Figure 5A’). Thus, local heterogeneity of eGFP transport exists in the cells, and, especially in the nucleus, regions with high eGFP concentration experience slower FL than regions with low eGFP concentration. Additional simulations confirmed that the local heterogeneity in FL, we detect by our approach is not due to detector noise or bad fitting performance (see Figures 4 and 5 and Additional file 1: Figure S6, S7 and S8). Note that we never observed stretched exponential intensity loss in the non-bleached compartment (i.e. C_1_) nor compressed FL in the bleached ROI (i.e. C_2_) in simulations of binding/barrier-dominated FLIP simulations. The fact, that we find this in FLIP experiments of eGFP in cells suggests that the simple compartment model is not adequate to describe local heterogeneity of intracellular eGFP motion. Further evidence for that is provided in the Additional files, especially Additional file 1: Figure S8. We also performed the opposite experiment and bleached repeatedly a small region in the nucleus (Additional file 1: Figure S9). As expected, fluorescence decayed more rapidly in the nucleus than in the cytoplasm in this experiment, since influx of eGFP into the nucleus is rate-limiting for FL in the cytoplasm. Moreover, we found that there is a positive correlation between amplitude and time constant map, as obtained from fitting the StrExp function to FL data (Additional file 1: Figure S9). Together, fitting the StrExp function on a pixel-by-pixel basis to binding/barrier-limited FLIP data allows for detecting regions of differing eGFP mobility in living cells.

### Heterogeneous transport of mutant huntingtin detected by quantitative FLIP analysis

**Figure 6:**
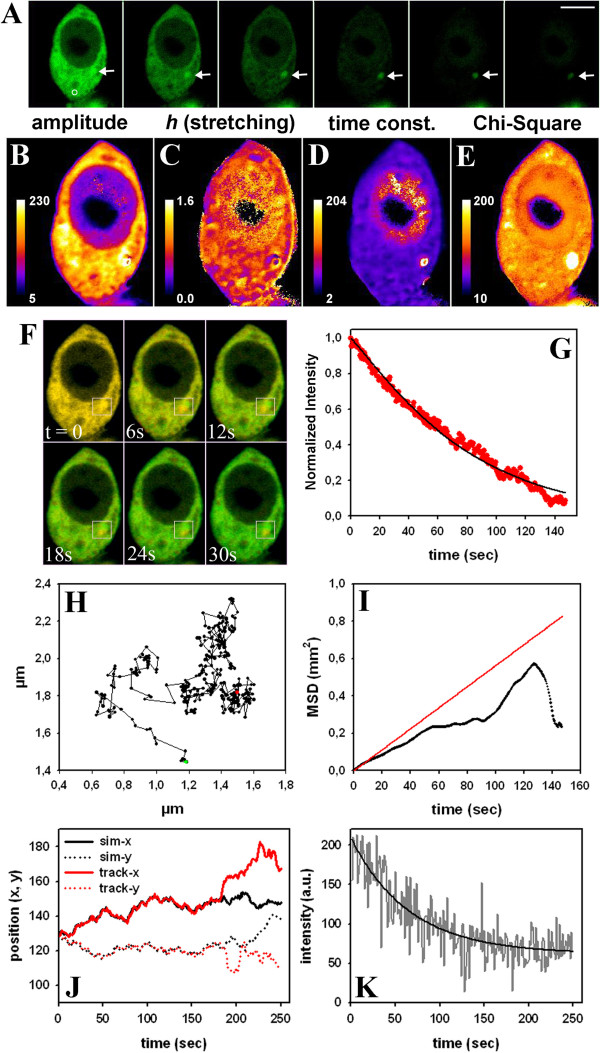
**Quantitative FLIP and single particle tracking of mobile IB’s in the cytoplasm.** A FLIP experiment with a bleach region of 10 pixels in diameter was performed in CHO cells expressing eGFP-Q73 on a temperature-controlled stage of a confocal microscope maintained at 35 ± 1°C. The total frame rate was 1.45 sec. **A**, montage of every 20^th^ frame of the data. Arrows point to an inclusion body (IB) in the cytoplasm. **B-E**, pixel-wise fit of the data to a StrExp function with **B**, amplitude map; **C**, map of the stretching parameter; **D**, time constant map; E, χ^2^-values showing the quality of the regression. All values are color-coded using a FIRE-LUT, and the range is indicated by the respective color bar. The time constant is given in seconds; all other values are without units. F, first frame of the time series is shown in green, while selected subsequent frames are overlayed in red (box in F shows movement of the IB). **G**, FL of eGFP-Q73 in the IB (red symbols) with fit to the StrExp function (black line). **H**, x,y-plot of the trajectory of the IB during the FLIP experiment. I, mean square displacement (MSD) calculated from the trajectory and linear fit to the five intital data points (red line). **J**, **K**, simulation and tracking of a particle experiencing FL with a rate constant, *k* = 0.01 sec^-1^. **J**, trajectory separated in x- (straight lines) and y-direction (dotted lines) for a particle undergoing bleaching (black lines, simulated trajectory; red lines, tracked trajectory). The tracked trajectory coincided with the simulated trajectory until to 180 sec (or frames). **K**, intensity of the tracked particle (grey lines) compared to a mono-exponential fit with residual. Bar, 5 μm.

**Figure 7:**
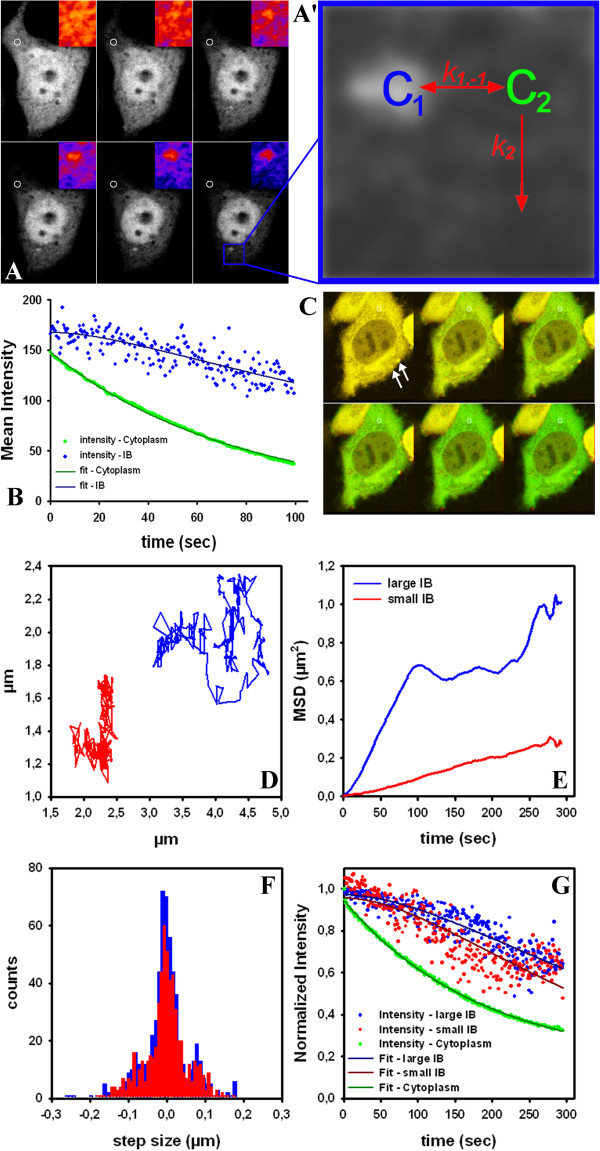
**Multi-compartment modeling of FLIP data reveals dynamics of eGFP-Q73 in inclusion bodies.** A FLIP experiment was performed in CHO cells expressing eGFP-Q73 as described in legend to Figure 6. **A**, montage of every 20^th^ frame of the data. Inset is a zoom of the small box pointing to an IB; a FIRE LUT is used for visualization purposes. A’, sketch of the multi-compartment (MC) model used for determining binding/dissociation parameters. **B**, FL kinetics for the IB (blue dots) and for a region in the cytoplasm (green dots) with overlayed fit to the MC model for the IB (compartment 1; dark blue line) and for the cytoplasm (compartment 2; dark green line). **C**, another FLIP sequence imaged with a total frame rate of 2.4 sec. First frame of the time series is shown in green, while selected subsequent frames are overlayed in red. This color coding visualizes movement of two IB’s at the cell edge (large one up and small one below; arrows). Tracking of the mobile IB’s and measurement of their intensity was performed using the SpotTracker plugin for ImageJ [47]. **D**, x,y-plot of the trajectories of the IB’s; **E**, mean square displacement (MSD) calculated from the trajectories; **F**, step length distribution between subsequent steps; **G**, fluorescence intensity of eGFP-Q73 in the IB’s (blue dots = large IB; red dots = small IB) and in the cytoplasm (green dots) and fit with the MC model for the IB’s (compartments 1, dark blue and red line, respectively) as well as for the cytoplasm (compartment 2, dark green line).

A group of neurodegenerative disorders is characterized by expansion of poly-glutamine (polyQ) repeats in aggregation prone proteins. For example, more than 36 polyQ repeats in mutant huntingtin (mtHtt) causes aggregation of the protein in the cytoplasm of affected cells causing Huntington disease [41]. There exist at least three populations of mtHtt in affected cells; monomers, oligomers and large aggregates called inclusion bodies (IB’s). Probably oligomers are the most cytotoxic form, but the exact mechanism causing Huntington disease is not understood and a matter of debate [42]. Formation of mtHtt aggregates has been studied in living cells by a variety of methods including FRAP and FLIP, photoactivation, Förster resonance energy transfer (FRET), fluorescence correlation spectroscopy (FCS) and Number & Brightness (N&B) analysis [42-46]. We transiently expressed eGFP-tagged constructs of control huntingtin (containing 23 glutamine (Q) repeats; eGFP-Q23) or mtHtt containing 73 repeats (eGFP-Q73) in Chinese hamster ovarian (CHO) cells. While eGFP-Q23 had similar characteristics in FLIP experiments as pure eGFP (not shown), eGFP-Q73 differed significantly (Figures 6 and 7). That protein forms large IB’s in the cytoplasm, which were identified based on their delayed FL kinetics (Figure 6A, arrows). Fitting the StrExp function on a pixel-by-pixel basis to the FL of that cell revealed some interesting aspects: firstly, nuclear FL was very slow (Figure 6D), probably caused by small aggregates which cannot pass the nuclear pore complex. Secondly, the map of the stretching parameters (Figure 6C), time constants (Figure 6D) and Chi-square values were more structured than observed in a similar experiment in cells only expressing eGFP (Figure 4) or cells expressing eGFP-Q23 (not shown). This is probably due to small protein aggregates moving in the cytoplasm; and finally the quality of the regression was significantly lower at the location of the IB, as inferred from the Chi-Square map (Figure 6E). The lower fit performance is a consequence of movement of the IB causing abrupt changes in intensity at the respective pixel positions during the FLIP experiment. This creates large artifacts in the pixel-wise fitting performance (Figure 6F visualizes the movement of the perinuclear IB). Accordingly, fitting the StrExp function to FL on a pixel-by-pixel basis is not appropriate for analyzing moving particles. We therefore combined quantitative FLIP with single particle tracking (SPT) performed with our SpotTracker plugin to ImageJ. This plugin not only provides particle coordinates but also the associated particle intensity [47]. We fitted FL measured for the tracked IB to a StrExp function to quantify the decay (Figure 6G). The parameters were *A* = 0.9455, *h* = 0.7876 and *τ* = 57.8 sec. In parallel, SpotTracker provides the IB trajectory (Figure 6H) from which we calculated the mean square displacement (MSD) of the moving IB (Figure 6I, black line). By a linear fit to the first 5 data points, we estimated an initial diffusion constant of D = 1.4x10^-3^ μm^2^/s (red line in Figure 6I). The MSD-plot deviated significantly from a linear function for large time steps excluding further analysis based on simple Brownian diffusion. We verified in simulated time-lapse sequences that the tracking algorithm could exactly determine particle positions, even for strongly decaying intensity and high noise levels (Figure 6J, K) [47]. FL was simulated with a mono-exponential process of rate constant k = 0.01 sec^-1^. Our tracking program 'SpotTracker' could reliably extract the FL kinetics: a fit to the extracted FL profile recovers the bleach rate constant approximately, (fitted *k*_b_ = 0.0156 sec^-1^) and the residual term is a good measure of the background noise (~60 intensity units). For these tests, we implemented random walk simulations in ImageJ using self-programmed macros. Particle bleaching during movement was simulated using a single exponential decay of intensity in the presence of noise, and the intensity value was updated for every new position along an image sequence. Since ImageJ by default allows only for generating random numbers from a uniform distribution, the Box-Muller method was implemented to transform these random numbers to a normal (Gaussian) distribution [48].

### Multi-compartment (MC) modeling of eGFP-mtHtt exchange between cytoplasm and IB’s

Although we were able to quantify FL of eGFP-mtHtt in moving IB’s using the strategy outlined above, this method does not provide cell-independent measures of eGFP-mtHtt dynamics. This is, since the FL depends not only on the protein exchange dynamics but also on the total cellular pool size of eGFP-mtHtt. In other words, for a larger cell the same FLIP imaging settings would give slower FL from a given IB than for a smaller cell, simply because it would take longer to deplete the whole protein pool in the larger cell. To directly compare exchange dynamics of eGFP-mtHtt between cytoplasm and IB’s from various cells, we developed an analytical MC model providing association and dissociation rate constants (see Figure 7 and Appendix 2). The basic idea of that modeling strategy is that FL kinetics in the IB’s becomes weighted by that in the cytoplasm for each cell thereby creating a cell-independent measure of protein exchange dynamics in the aggregates. For that MC model, we assumed that eGFP-mtHtt is distributed in two pools; several small IB’s as the first pool and the large cytoplasm as the second pool. Exchange between the IB’s (compartment(s) 1, C_1_) and the cytoplasm (compartment 2, C_2_) takes place with rate constants *k*_1_ and *k*_−1_ (Figure 7A’). The number of the IB’s can be large as long as they together contain a much smaller amount of eGFP-mtHtt than the cytoplasmic compartment. Furthermore, the experimental conditions should be chosen such that binding-limited FLIP conditions are established (i.e., no depletion zone like in diffusion-limited FLIP experiments; this can be verified by pixel-wise fitting the StrExp function to FLIP image sets). Finally, it is presumed that the cytoplasmic pool of eGFP-mtHtt changes only because of the bleach but not due to release of eGFP-mtHtt from IB’s. These conditions are justified by the large size of the cytoplasmic compared to the IB pool (see above), and they allow for decoupling the differential equation system (see Appendix 2 for further details). We first fitted the FL in the cytoplasm caused by the repeated bleaching with a mono-exponential decay function (Figure 7B; green symbols, data; dark green line, fit). Next, we used the estimated rate constant as input for fitting FL in the IB outlined by the blue box in Figure 7A (Figure 7B shows FL data as blue symbols and the fit as dark blue line).The model fitted the experimental data very well (R^2^ > 0.98) and gave a half-time for FL in the cytoplasm of t_1/2_= 51.7 sec. Note that the FL in the cytoplasm is solely a consequence of the bleach protocol. In contrast, the FL in the IB is a result of both, the dissociation of eGFP-Q73 from that protein aggregate and the subsequent photodestruction of the released eGFP-Q73 in the bleach ROI. Using the FL kinetics in the cytoplasm (see above) and the MC model, eGFP-mtHtt dissociation could be calculated to occur here with a half time of t_1/2_= ln2/*k*_1_ =73.8 sec. Another example of a cell with 2 IB’s of varying mobility is shown in Figure 7C-G. The larger IB is more mobile but its associated eGFP-Q73 monomers are in slightly slower exchange with the cytoplasm than for the smaller IB (i.e., half-time of dissociation was t_1/2_= 200.6 sec and t_1/2_= 142.3 sec for the large and small IB, respectively). We tracked additional IB’s in the cytoplasm of several cells and found mean off- and on-rate constants for eGFP-Q73 of *k*_1_ = 0.0127 ± 0.004 sec^-1^ and *k*_*−*1_ = 0.016 ± 0.006 sec^-1^ (n=6; mean ± SE). Thus, the average half-time of eGFP-Q73 dissociation from IB’s is 101.2 ± 30 sec in our experiments. The observed heterogeneity in exchange dynamics of eGFP-mtHtt between cytoplasm and IB’s is in line with earlier qualitative FLIP and FRAP experiments [43,44]. Together, combined fitting of FL kinetics to the MC model and SPT of moving IB’s enabled us to determine in parallel aggregate mobility and exchange dynamics of mtHtt between moving aggregates and the cytoplasm.

## Discussion

Fluorescence loss in photobleaching (FLIP) is a dynamic imaging technique with the potential to include spatial information into analysis of protein dynamics, but this potential has not been explored. By treating images as data arrays rather than pictures, we present two FLIP analysis methods for assessing intracellular transport dynamics of fluorescent proteins. Our first approach comprises fitting a StrExp function to FL kinetics on a pixel-by-pixel basis. The rationale behind this idea is that the time-dependent rate coefficient of the StrExp function is suitable to describe transport under conditions, where the “well-stirred compartment” assumption fails. Diffusion gradients or topological constraints cause deviation from the concept of compartment homogeneity, and that can be modelled with differential equations having time-dependent rate coefficients [35]. The StrExp function is based on such an equation, and our method of pixel-wise fitting this function to FLIP image sets provides kinetic maps of locally heterogeneous transport and allows for exact data reconstruction using the derived model parameters (for example, see Figure 4A). FLIP as an imaging method, has the advantage that the user can determine the time resolution of the experiment by setting pauses of arbitrary length between individual bleaches. For short pauses, i.e., fast repeated bleaching; FLIP-experiments might become diffusion-limited (see Figures 1, 2 and 3 and Additional file 1: Figure S1, S2 and S3). We demonstrate on simulated and experimental FLIP image sets that a spatial gradient of the fitting parameters (i.e., stretching, *h*, and time or rate constant, respectively) of the StrExp function is characteristic for a diffusion-limited FLIP experiment.

For larger pauses between the bleaches, eventual hindrance to diffusion due to transient binding or barriers can be detected on the sub-cellular level by pixel-wise FLIP analysis without pre-selection of ROIs (Figures 4 and 5). This result is not in line with isotropic normal diffusion of eGFP in cells, though eGFP is known to show minimal interaction with its surroundings [49]. An often (but not always, see [50]) overlooked aspect in the literature of biophysical diffusion studies (for example using FRAP) is that not only homogenous diffusion but also locally heterogeneous diffusion (i.e., with a space-dependent diffusion constant) with continuity between the regions of varying D will cause an equilibration of any concentration gradient. This is illustrated in the simulation shown in Figure 2 (see above). Thus, already the initial distribution of eGFP in the prebleach image tells us that crowding effects exclude eGFP from some regions in the nucleus and the cytoplasm. We are aware of one recent study, where the structured fluorescence intensity of an inert fluorescent protein (i.e., yellow fluorescent protein, YFP) in a single image of the cell has been ascribed to the cytoplasm and nucleoplasm being an ‘effective porous medium’ [50]. In porous media, one distinguishes diffusion in the liquid phase (here the cytosol or nucleosol) from that in the medium with constrained motion. We speculate that the large variation in rate coefficients, we find for FLIP data of eGFP in the cytoplasm is a consequence of this porosity. Since the optical resolution of the confocal microscope (i.e., about 250 nm) is several times larger than the pore size of the cytoplasm (i.e., 30–100 nm), one cannot segment this cytoplasmic ultrastructure, though it obstructs protein motion [51]. Furthermore, even for ‘inert’ biomolecules lacking specific interactions, like eGFP, not only obstruction is found but also enhancement of diffusion could be triggered by active transport of other components causing enforced motor activity and dynamic remodelling of the cytoskeleton network [52]. Thus, the cytosol might provide rapid decay channels for eGFP, while the high protein concentration, organelles and cytoskeleton constrain eGFP mobility otherwise. Rapid decay channels can be detected with the StrExp fit to FLIP data as individual pixels with high rate coefficients, even far from the bleached ROI (see Figures 4 and 5 and Additional file 4: Movie S3). Additional simulations of binding-barrier-limited FLIP experiments using the compartment model of Equations. 3–5 but with rate constants varying on a pixel-by-pixel basis were performed to account for spatial heterogeneity (Additional file 1: Figures S7 and S8). By fitting the StrExp function to these simulated time courses, we found local variation of the recovered parameters (i.e. *h*-map and time constant map), but in a much narrower range than observed for the FLIP data for eGFP in cells (compare Figure 4 and 5 with Additional file 1: Figure S8). Thus, transport of eGFP as measured by FLIP follows a complex dynamic relaxation pattern due to local hindrance to diffusion on one hand and local enhancement of diffusive transport on the other. Such complex behaviour cannot be understood from a simple binding-barrier or diffusion model of intracellular transport but it can be revealed using pixel-wise fitting of the StrExp function to FLIP image sets.

Using our quantitative FLIP analysis, we could also visualize local diffusion barriers for eGFP in the nucleus (see Figures 4, 5 and Additional file 1: Figure S9). These kinetic domains in the nucleus are likely a consequence of the fractal chromatin organization [53]. Recent results published by Gratton and co-workers detect also barriers for eGFP diffusion in the nucleus of CHO cells using pair correlation functions (pCF) [54]. An advantage of their method is the ability to extract local diffusion coefficients directly from the cross-correlation of intensity fluctuations of the same fluorophore at two different but adjacent points in the sample [55]. Rare intensity bursts report on eGFP molecules travelling across regions with strongly varying DNA density [54]. The pCF method has also been applied to study transport of eGFP between nucleus and cytoplasm, where the nuclear membrane including the NPC appeared as obstacle to eGFP inter-compartment diffusion [56]. This diffusion barrier is nicely detected in the time constant map of our quantitative pixel-based FLIP analysis (see Figure 4 G, J), suggesting that fluorescence fluctuation techniques like pCF and our approach provide complementary information.

To the best of our knowledge, there is only one full publication published this year, which also applies a pixel-wise regression of a decay function to FL data of FLIP image sets [23]. Lelieveldt and colleagues assumed a simple exponential decay of the FL allowing for linear regression in the logarithmic space. They also performed a noise reduction as pre-processing step and were able to correct for sudden motion of the specimen or the image field during acquisition of the FLIP data [23]. Our approach implemented in ‘PixBleach’ uses a Gaussian filtering in the space and time domain as pre-processing step which corrects in our hands sufficiently for image noise and for small movements (in a range of one or two pixels) [40]. Since we apply a motion correction as pre-processing step in case of larger displacement, no further corrections were necessary for the image data used here (see Methods section and [57]). Using the StrExp function is clearly superior to simple mono- or bi-exponential decay models, since it can model FL inside and outside the bleached region for both, binding/barrier- and diffusion-limited FLIP experiments. The delayed decay described by a compressed exponential function as a consequence of binding or barriers (in binding/barrier -limited FLIP experiments) or due to long distance to the bleached area (in diffusion-limited FLIP experiments) cannot be adequately described with simple exponential decay functions (see Table 1).

Quantitative FLIP microscopy should also be a method to compare results from different cells. This, however, is not possible with the pixel-based FLIP analysis, because the kinetic parameters recovered from the StrExp fit to data are a function of the total amount of fluorophore in a given cell, the diameter of the bleach spot and the length of the pauses between bleaches. The empirical StrExp fitting function only provides information about the nature of the observed transport process (e.g. diffusion- or binding-limited, see Table 1) and about local variation in transport dynamics for a given experiment and cell. Experiment-independent binding and dissociation parameters of a fluorescent protein can therefore not be measured by this approach. To overcome this limitation, we developed a complementary method based on compartment modelling of FLIP data and applied it to determine local heterogeneity in intracellular protein aggregation. Such aggregation is observed in cellular models of various neurodegenerative diseases [45,58,59]. Since protein aggregates tend to move during FLIP experiments, we combine tracking of individual IB’s containing mtHtt with extended polyQ repeat (eGFP-Q73) with non-linear regression of an analytical MC model to the FLIP-induced intensity decay in these structures (Figures 6, 7). This enables us to measure mobility parameters, like diffusion constants from the calculated MSD and to determine in parallel association and dissociation rate constants for eGFP-mtHtt from the measured FL kinetics in IB’s. Thus, we demonstrate that mtHtt in IB’s can exchange with mtHtt in the cytoplasm in agreement with earlier observations for other polyQ diseases [43]. Using our MC modeling strategy, we can derive for the first time in-vivo binding parameters of eGFP-mtHtt showing that the exchange takes place with a half-time of 2–4 min. Since MC modeling of FLIP data provides physical parameters, this method will be of high value for quantitative comparison of protein aggregates in Huntington disease with that in other polyQ diseases, like various forms of ataxia. In fact, compressed exponential FL, similar as we found for eGFP-mtHtt has been reported in FLIP-experiments of ataxin-3 aggregates, in the nucleoplasm of COS7 cells [58]. We are aware of the fact, that the presented two modelling approaches for FLIP data analysis are based on somehow contradictive assumptions: pixel-wise fitting reveals local heterogeneity of protein transport in various cellular pools, while MC modelling of FLIP experiments requires the assumption of well-mixed compartments. Solving this contradiction satisfactorily would require modelling the whole spatiotemporal protein dynamics including cell geometry, space-dependent diffusion constants, diffusion barriers, moving entities etc., but this is too ambitious at the moment and outside the scope of this article. We found that deviation of FL kinetics from a mono-exponential decay becomes negligible when larger cytoplasmic regions (above ~5 pixels) are included in the analysis (not shown). We also implemented a StrExp decay model for the FL in the bleached compartment (C_2_) in our MC model, but that recovered a mono-exponential decay with *h* = 1 (not shown, but see Figure 7C, green symbols and line). Together, with the observed time hierarchy, this makes the assumption of a well-mixed cytoplasmic compartment compared to measured FL of eGFP-Q73 in IB’s reasonable.

## Conclusions

We present two new approaches for quantitative analysis of FLIP experiments in living cells. Pixel-wise fitting of a StrExp function to FLIP image sets allows for detecting areas of different probe mobility, while physical modelling of FLIP data in the second method provides for the first time dissociation parameters of fluorescent proteins from moving entities. Our methods are easy to apply to other transport problems, where a fluorescent biomolecule is soluble in the nucleus or cytoplasm and binds to or partitions into static structures (for pixel-based FLIP quantification) or dynamic structures (for combined SPT and MC modeling). Typical other applications could be transient binding of ras-protein to the Golgi apparatus and plasma membrane [60], of lipases and other proteins to lipid droplets [61], cohesin mobility in the nucleus of yeast cells [62], recruitment of rab proteins to endosomes [63] or the dynamic partitioning of fluorescent drugs and lipids into subcellular membranes [64].

## Methods

### Reagents and cell culture

Fetal calf serum and DMEM were from GIBCO BRL (Life Technologies, Paisley, Scotland). 3,3,3’,3’-tetramethylindocarbocyanine perchlorate (DiIC12) was purchased from Molecular Probes (Eugene, Oregon, USA). All other chemicals were from Sigma Chemical (St. Louis, MO). Medium 1 contained 150 mM NaCl, 5 mM KCl, 1 mM CaCl_2_, 1 mM MgCl_2_, 5 mM glucose and 20 mM HEPES (pH 7.4). McArdle RH7777 (McA) cells expressing enhanced green fluorescent protein (EGFP) have been reported previously [65]. These cells were grown in DMEM with 4.5 g/l glucose, supplemented with 10% heat-inactivated FCS and antibiotics. Chinese hamster ovarian (CHO) cells were purchased from ATCC (http://www.atcc.org; LGC Standards Office Europe, AB, Boras, Sweden) and grown in bicarbonate buffered Ham’s F-12 medium supplemented with 5% FCS and antibiotics. Cells were routinely passaged in plastic tissue culture dishes. Two to three days prior to experiments, cells were seeded on microscope slide dishes coated with poly-D-lysine.

### Confocal laser scanning fluorescence microscopy

Confocal microscopy was performed using a laser scanning inverted fluorescence microscope (Zeiss LSM 510 META, Zeiss, Jena, Germany) equipped with a 63x 1.4 NA plan Apochromat water immersion objective and a 37°C temperature control (Zeiss, Jena, Germany). Fluorescein and eGFP fluorescence was collected with a 505–530 bandpass filter after excitation with a 25-milliwatt argon laser emitting at 488 nm. FLIP experiments were performed by first defining regions of interest (ROI), which were repeatedly bleached, while an image was acquired with reduced laser power (0.5% output) at the start of the experiment and after each bleach. Either one iteration (for eGFP-Huntingtin constructs in CHO cells) or five iterations (for eGFP in McA cells) with 100% laser power were used for the bleaching pulses. An eventual pause between the bleaches ensured some recovery in the ROI. Images were acquired using the time-lapse function of the Zeiss LSM510 Meta confocal system. The microscope was located at a nitrogen-floated table to prevent vibrations and focus drift and contained a temperature-controlled stage maintained at 35 ± 1°C. For spatial registration of image stacks, a plugin to ImageJ named “StackReg” developed by Dr. Thevenaz at the Biomedical Imaging Group, EPFL, Lausanne, Switzerland was used [57]. One pixel typically corresponded to 0.055 × 0.055 μm (for example in Figures 4 and 5).

### Non-linear regression of a stretched exponential function to experimental and synthetic FLIP data

Pre- and post-bleach images were imported into ImageJ and combined into a single stack in 16-bit format. Image time series were smoothed with a Gaussian filter (standard deviation = 0.5) in the spatial and temporal domain. Fluorescence loss *I*($\vec{r}$, *t*) at each pixel position $\vec{r}$ = (*x*, *y*) due to repeated bleaching of fluorescence in the selected ROI was fitted to a stretched exponential of the form:

(1) $$\begin{array}{l} I\left(\vec{r},t\right)=I_{0}\left(\vec{r}\right)\cdot \exp\left[-{\left(\frac{t}{\tau \left(\vec{r}\right)}\right)}^{\frac{1}{h\left(\vec{r}\right)}}\right]\\ \;+I_{\infty }\left(\vec{r}\right) \end{array}$$

using “PixBleach” our recently developed image fitting program implemented in ImageJ software (download at: http://bigwww.epfl.ch/algorithms/pixbleach/) [40]. Here, *I*_0_ is the pre-bleach intensity of the dynamic fluorescence pool, *τ* is the decay time constant and *I*_∞_ is the remaining intensity at infinite time, resembling either an immobile fraction or autofluorescence of the cells (most often, *I*_∞_ approximates the background noise level). The decay time constant *τ* describes the rate of decrease in fluorescence intensity of the probe, either directly in the bleached region due to the intense laser beam, or outside the bleached region due to transport towards the repeatedly bleached ROI. Thus, the bleached regions act like a sink for fluorescent molecules being located outside the ROI. The stretched exponential differs from a normal mono-exponential function by an additional parameter, *h*, describing the stretching or compression of the decay compared to a mono-exponential function. This parameter is for *h* >1 a direct measure of the width of the decay constant distribution (see Results section). The stretched exponential model can therefore be considered as a linear superposition of simple mono-exponential decays [28]. All parameter maps generated by ‘PixBleach’ are given in 32-bit format and were used in that format for further calculations.

### Compartment modeling of fluorophore transport in FLIP experiments

In the classical FLIP experiment, a pause between the intended local photobleaching ensures that emitting flurophores are transported into the bleached area such that the recovery process is not diffusion-limited. In this case and if transient binding events take place, the FLIP experiment can be modeled by a set of ordinary differential equations (see below). The simplest model for transport and bleaching contains two compartments; (1) the region outside the bleached area from which transport to the bleached ROI occurs with the amount of fluorophores, *N*_1_(*t*) and (2) the repeatedly bleached region acting as a “fluorescence sink” for the transport of the amount of labeled molecules, *N*_2_(*t*). For the modelling, we assume that the concentration of fluorophores, *n*(*x*, *y*) = *N*/*V*, where *V* is the volume of the respective compartment, is proportional to the emitted fluorescence intensity at a given wavelength, λ, according to [66]:

(2) $$I\left(x,y,\lambda \right)=f\left(\theta \right)\cdot g\left(\lambda \right)\cdot \Phi_{F}\cdot L\cdot \varepsilon \cdot b\cdot n\left(x,y\right)$$

Here, *f*(*θ*) is a geometric factor, *g*(*λ*) is the wavelength-dependent quantum efficiency of the detector, *Φ*_*F*_ is the quantum yield of the fluorophore, *L* is the excitation intensity, *ε* is the molar extinction coefficient (in M^-1^ cm^-1^) and *b* is the optical path length. Thus, we can infer relative values for the concentration or density of probe molecules in certain cellular regions from the pixel-wise detected fluorescence in the images.

We consider bidirectional transport of fluorophores between compartment 1 and 2 with the forward and backward rate constants *k*_1_ and *k*_−1_, respectively. The bleaching process within the repeatedly bleached area (compartment 2) is modeled by the rate constant *k*_2_.

(3) $$N_{1}\overset{k_{1},k_{-1}}{↔}N_{2}\overset{k_{2}}{\to }$$

This gives the following system of differential equations:

(4ab) $$\begin{array}{l} \frac{dN_{1}}{dt}=-k_{1}\cdot N_{1}+k_{-1}\cdot N_{2}\\ \frac{dN_{2}}{dt}=k_{1}\cdot N_{1}-(k_{-1}+k_{2})\cdot N_{2} \end{array}$$

The time-dependent solutions for both compartments calculated from the eigenvalues λ_1_ and λ_2_ and corresponding eigenvectors of the associated kinetic matrix are:

$$N_{1}\left(t\right)=\frac{\left[\left(A+B\right)\cdot \exp\left(\lambda_{1}\cdot t\right)+\left(-A+B\right)\cdot \exp\left(\lambda_{2}\cdot t\right)\right]\cdot N_{1}^{0}}{2\cdot B}$$

$$N_{2}\left(t\right)=\frac{\left[\left(-A2+B\right)\cdot \exp\left(\lambda_{1}\cdot t\right)+\left(A2+B\right)\cdot \exp\left(\lambda_{2}\cdot t\right)\right]\cdot N_{2}^{0}}{2\cdot B}$$

with

(5af) $$\begin{array}{l} A=-k_{1}\cdot (N_{1}^{0}+N_{2}^{0})-k_{2}\cdot N_{2}^{0}\\ A2=k_{1}\cdot (N_{1}^{0}+N_{2}^{0})-k_{2}\cdot N_{2}^{0}\\ B=\sqrt{-4\cdot k_{1}\cdot k_{2}\cdot {\left(N_{2}^{0}\right)}^{2}+{\left(k_{1}\cdot \left(N_{1}^{0}+N_{2}^{0}\right)+k_{2}\cdot N_{2}^{0}\right)}^{2}}\\ \lambda_{1}=0.5\cdot (A-B)/N_{2}^{0}\\ \lambda_{2}=0.5\cdot (A+B)/N_{2}^{0} \end{array}$$

Here, the initial values *N*_1_^0^ and *N*_2_^0^ describe the initial amounts outside and inside the bleached region, respectively. We have eliminated *k*_−1_ by detailed balance, *k*_1_ · *N*_1_^0^ = *k*_− 1_ · *N*_2_^0^. This kinetic compartment model was simulated with varying parameter combinations as function of time using SigmaPlot 9.0 (SPSS Inc, Chicago, IL, USA) or as function of time and pixel coordinates using self-programmed Macros in ImageJ. An extension of that model to an arbitrary number of non-bleached compartments 1 is given in Appendix 2.

### Analytical model of FLIP experiments with spatially invariant diffusion coefficient

A FLIP experiment without pause between the individual bleaches results in diffusion-limited transport of fluorophores, *n*, towards the bleached area (see Figure 1A, B for geometry). This can be modeled using the following partial differential equation: $\frac{\partial n}{\partial t}=D\cdot \nabla^{2}n-k\cdot n\cdot X$ with

(6) $$X=\{\begin{array}{l} 1,\ldots r<r_{1}\\ 0,\ldots r>r_{1} \end{array}$$

with the boundary conditions that $n,\frac{\partial n}{\partial r}$ are continuous at *r* = *r*_1_ and $\frac{\partial n}{\partial r}=0$ at *r* = *r*_2._ The solution of this model is given in Appendix 1.

### Numerical simulation of FLIP experiments with spatially heterogeneous diffusion

It has been reported that diffusion coefficients can depend on the position within the cells (i.e., $D=D\left(\vec{r}\right)$ =*d* with $\vec{r}$ being the vector of particle positions [21]. For this situation, no analytical solution to Eq. 6 are available. We therefore performed a numerical simulation of a space dependent diffusion model for photobleaching rewriting Eq. 6 to:

(7) $$\frac{\partial n}{\partial t}=\mathrm{div}\left(d\mathrm{grad}n\right)-k\cdot n\cdot X$$

Here, *n* models the number density of fluorophores, as above, while the characteristic function X describes the cell area that is illuminated by the laser beam, as above. The bleaching rate is again *k*. The mathematical model, in variational form,

(8) $$\int \frac{\partial n}{\partial t}\Phi \;dx+\int d\mathrm{grad}n\cdot \mathrm{grad}\Phi \;dx+\int knΧ\Phi \;dx=0\;\forall \Phi$$

is easily implemented in FEniCS (http://www.fenicsproject.org). The FEniCS project DOLFIN compiles the pseudo-code and assembles the discrete finite element system. The resulting linear system is solved using the PETSc (http://www.mcs.anl.gov/petsc) toolkit for scientific computing. We simulated a cylindrical bleaching experiment on a disk with *r*_2_ = 1μm. A circular bleached area of radius with *r*_1_ = 0.5 μm was placed in the disk center. The bleaching rate is set to k = 10 sec^-1^ and the diffusion coefficient is d_L_ = 0.2 μm^2^/sec and d_R_ = 0.8 μm^2^/sec on the left and right half circle, respectively.

## Competing interests

The authors declare that they have no competing interests.

## Authors' contributions

Conceived and designed experiments: DW. Performed experiments: DW, LMS, FWL. Analyzed experimental data: DW, FWL. Developed mathematical models and performed simulations: DW, DS, HJS, MAL. Performed non-linear regression to simulated data sets: DW. Wrote the paper: DW (with comments and criticism from all authors). All authors read and approved the final manuscript.

## Appendix

### Appendix 1

The diffusion problem in Equation 6 reads in Laplace space ($n\left(r,u\right)=\int_{0}^{\infty }n\left(r,t\right)\cdot e^{-ut}dt$):$\begin{array}{l} u\cdot n-n_{0}=D\cdot \nabla^{2}n-k\cdot n\\ u\cdot n-n_{0}=D\cdot \nabla^{2}n \end{array}$ for $\begin{array}{l} r<r_{1}\\ r>r_{1} \end{array}$where *n*_0_ is the initial density and

$$\nabla^{2}=\frac{1}{r}\cdot \frac{\partial }{\partial r}\cdot r\cdot \frac{\partial }{\partial r}$$

It has the solution$\;n=B_{1}\cdot I_{0}\left(\sqrt{\frac{u+k}{D}}\cdot r\right)+\frac{n_{0}}{u+k}$ for *r* < *r*_1_

$$n=A_{2}\cdot K_{0}\left(\sqrt{\frac{u}{D}}\cdot r\right)+B_{2}\cdot I_{0}\left(\sqrt{\frac{u}{D}}\cdot r\right)+\frac{n_{0}}{u}$$

for *r* > *r*_1_ where *I*_*n*_,*K*_*n*_represent modified Bessel functions of the first and second kind, respectively of order *n*, and *B*_1_,*A*_2_, *B*_2_ are defined from the boundary conditions (see main text and Eq. 6). We get

$$B_{1}=-\sqrt{\frac{u}{u+k}}\;\cdot \frac{I_{1,1}^{0}\cdot K_{1,2}^{0}-I_{1,2}^{0}\cdot K_{1,1}^{0}}{Y}\cdot \frac{k\cdot n_{0}}{u\left(u+k\right)}$$

$$B_{2}=-\frac{I_{1,1}^{k}\cdot K_{1,2}^{0}}{Y}\cdot \frac{k\cdot n_{0}}{u\left(u+k\right)}$$

$$\begin{array}{l} Y=I_{0,1}^{0}\cdot I_{1,1}^{k}\cdot K_{1,2}^{0}\\ \;+\sqrt{\frac{u}{u+k}}\;\cdot \left[I_{0,1}^{k}\cdot K_{1,1}^{0}\cdot I_{1,2}^{0}-I_{0,1}^{k}\cdot I_{1,1}^{0}\cdot K_{1,2}^{0}\right]\\ \;+K_{0,1}^{0}\cdot I_{1,2}^{0}\cdot I_{1,1}^{k} \end{array}$$

$$A_{2}=-\frac{I_{1,1}^{k}\cdot I_{1,2}^{0}}{Y}\cdot \frac{k\cdot n_{0}}{u\left(u+k\right)}$$

where $X_{n,m}^{k}=X_{n}\left(\sqrt{\frac{u+k}{D}}\cdot r_{m}\right)$ with *X* = *K*, *I*A numerical inverse Laplace transform has been used to generate solutions in real space with Mathematica Version 7.0 (Wolfram research Inc.).

### Appendix 2

A multi-compartment model for binding-limited FLIP experiments can be described by a scheme like

$$N_{1}^{i}\overset{k_{1}^{i},k_{-1}^{i}}{↔}N_{2}\overset{k_{2}}{\to },$$

where *i* numbers individual compartments of type 1 embedded in a large compartment labeled 2. This gives the following system of differential equations:

$$\begin{array}{l} \frac{dN_{1}^{i}}{dt}=-k_{1}^{i}\cdot N_{1}+k_{-1}^{i}\cdot N_{2}\\ \frac{dN_{2}}{dt}=\sum_{i}k_{1}^{i}\cdot N_{1}^{i}-(\sum_{i}k_{-1}^{i}+k_{2})\cdot N_{2} \end{array}$$

We assume that compartment 2 is much larger than the individual compartments 1, such that FL in compartment 2 is solely governed by rate constant k_2_. This gives for FL in compartment 2:

$$N_{2}\left(t\right)=N_{2}^{0}\cdot \exp\left(-k_{2}\cdot t\right)$$

The dynamics of the N_1_^i^(t) is assumed to satisfy

$$\frac{dN_{1}^{i}}{dt}=-k_{1}^{i}\cdot N_{1}\left(t\right)+k_{-1}^{i}\cdot N_{2}^{0}\cdot \exp\left(-k_{2}\cdot t\right)$$

By taking into account that

$$k_{-1}^{i}=k_{1}^{i}\cdot N_{1}^{i,0}/N_{2}^{0}$$

with the initial amount in the ith compartment of type 1 denoted as N_1_^i,0^, we obtain the following solution

$$N_{1}^{i}\left(t\right)=N_{1}^{i,0}\cdot \left(\frac{k_{1}^{i}}{k_{1}^{i}-k_{2}}\cdot \exp\left(-k_{2}\cdot t\right)-\frac{k_{2}}{k_{1}^{i}-k_{2}}\cdot \exp\left(-k_{1}^{i}\cdot t\right)\right)$$

The solution N_2_(t) was fitted to FL in the cytoplasm. The solution for the N_1_^i^(t) was fitted separately to each IB per cell using the value of *k*_2_ obtained from the fit of N_2_(t) to FL in the cytoplasm, such that the first IB in a cell gives k_1_^1^ and N_1_^1^(t), the second k_1_^2^ and N_1_^2^(t), and so on.

## Supplementary Material

Additional file 1 **Figure S1.** Simulation of the StrExp function and distance-dependence of fitting to homogenous diffusion. Figure S2. Bleach profile of the Argon laser at 488 nm for various objectives. Figure S3. 3D simulation of a FLIP experiment with heterogeneous diffusion in the unit sphere. Figure S4. FLIP experiment of BODIPY-cholesterol in CHO cells and fitting with the StrExp function. Figure S5. Simulation of the homogeneous compartment model and fitting with the StrExp function. Figure S6. Effect of additive image noise on fitting performance. Figure S7. Effect of time noise on fitting performance. Figure S8. Correlation between amplitude and time constant maps in the StrExp fit to the spatially Figure S9. Pixel-wise FLIP analysis of eGFP in the nucleus.heterogeneous model. Click here for file

Additional file 2 **Movie 1.** Numerical simulation of FLIP experimentwith space-dependent diffusion coefficient. Click here for file

Additional file 3 **Movie 2.** Time course of barrier-limited FLIPexperiment of eGFP. Click here for file

Additional file 4 **Movie 3.** Time evolution of rate coefficients forbarrier-limited FLIP experiment of eGFP. Click here for file
