# Supplementary material for: Quantitative fluorescence loss in photobleaching for analysis of protein transport and aggregation
Source: BMC Bioinformatics. 2012 Nov 13;13:296. doi: 10.1186/1471-2105-13-296 (PMC3557157; doi:10.1186/1471-2105-13-296)
Supplement: Additional file 1 — Figure S1. Simulation of the StrExp function and distance-dependence of fitting to homogenous diffusion. Figure S2. Bleach profile of the Argon laser at 488 nm for various objectives. Figure S3. 3D simulation of a FLIP experiment with heterogeneous diffusion in the unit sphere. Figure S4. FLIP experiment of BODIPY-cholesterol in CHO cells and fitting with the StrExp function. Figure S5. Simulation of the homogeneous compartment model and fitting with the StrExp function. Figure S6. Effect of additive image noise on fitting performance. Figure S7. Effect of time noise on fitting performance. Figure S8. Correlation between amplitude and time constant maps in the StrExp fit to the spatially Figure S9. Pixel-wise FLIP analysis of eGFP in the nucleus.heterogeneous model. [file 1471-2105-13-296-S1.pdf]

Supporting information

Mathematical description of the stretched exponential function

The stretched exponential model with $h > 1$ can be considered as a linear superposition of simple mono-exponential decays according to (*1*):

$$I(t) = \int_0^\infty \rho(k) \cdot e^{(-k \cdot t)} dk \qquad \text{(S1)}$$

, where $\rho(k)$ is the continuous distribution of decay rate constants and $I(0) = 1$.

The function ρ(*k*) is the inverse Laplace transform of *I*(*t*) and resembles, for $\int_0^\infty \rho(k)dk = 1$ the distribution (or probability density function, PDF) of the kinetic rate constants, k_i, also called the eigenvalue spectrum of the underlying kinetic matrix (*2*). The distribution of rate constants, rather than a single rate constant, as in a simple mono-exponential decay, is a consequence of the underlying differential equation with time-dependent rate constant *k*(*t*), called a rate coefficient (*2*):

$$\frac{dn}{dt} = -k(t) \cdot n \qquad \text{(S2)}$$

Here, *n* is the decaying species, for example number of fluorophores in a given volume with start amount n_0. The normalized decay law is thus

$$I(t) = \frac{n(t)}{n_0} = e^{-\left(\frac{t}{\tau_0}\right)^{1/h}} \qquad \text{(S3)}$$

and, thus *k*(*t*) gets:

$$k(t) = -\frac{d(\ln I(t))}{dt} = \frac{1}{h \cdot \tau_0} \cdot \left(\frac{t}{\tau_0}\right)^{\frac{1}{h}-1} \qquad \text{(S4)}$$

We plotted the right side of Eq. S4 for τ_0 = 8.3 sec for varying values of the parameter h in Fig. S1B. One can clearly see that $k(t)$ increases as function of time for h < 1, stays constant for h = 1 and decreases for h > 1. This is in accordance with an accelerating, constant, and slowing "reaction-speed" for the compressed, mono-exponential and stretched exponential case, respectively (see above). For h > 1, $k(t=0) \rightarrow \infty$, as can be seen from inspection of the right side of Eq. S4 and from Fig. S1B. Thus, in principle, the rate coefficient is infinite for h > 1 at the start of the time course. Note also that the eigenvalue spectrum, $\rho(k)$, is not a PDF for the compressed case, since it takes positive and negative values (*2*).

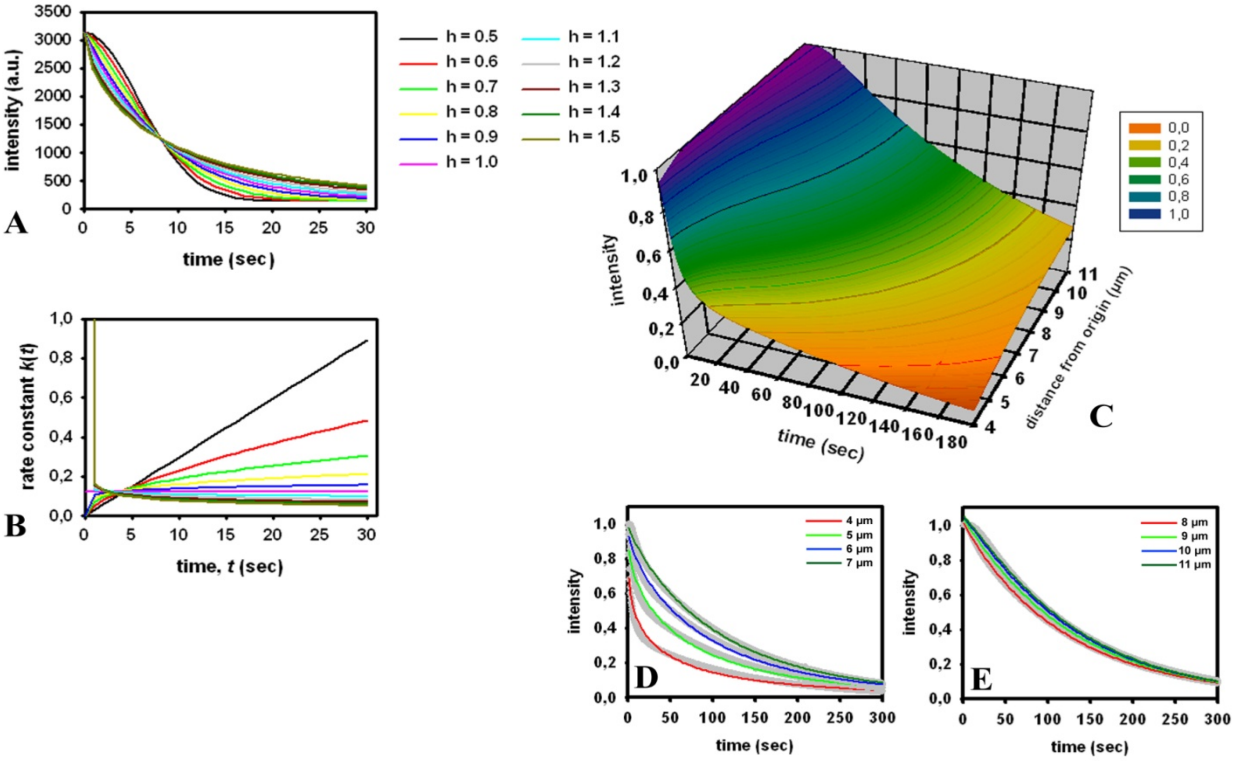

Fig. S1. Simulation of the StrExp function and distance-dependence of fitting to homogenous diffusion
A, The stretched exponential function according to Eq. 1 was plotted for one arbitrary position r with prebleach intensity I_0 = 3000 (a.u.), τ_0 = 1/k = 8.3 sec and stretching parameter h ranging from h = 0.5 … h = 1.5, respectively. A delayed onset of intensity decay up to τ is observed for h < 1 resembling the compressed exponential case. For h > 1 (the stretched exponential case) the fluorescence decay is faster than exponential before τ, and slower afterwards. B, The time-dependent rate constant (rate coefficient) of Eq. S4 was plotted for the same parameter combination as in A. We find that $k(t)$ increases as function of time for h < 1, stays constant for h = 1 and decreases for h > 1 reflecting accelerating, constant, and slowing "reaction-speed" for the compressed, mono-exponential and stretched exponential case, respectively (see main text for further explanations and applications below). C, 3D-plot of the simulated homogenous particle diffusion model with D = 1 μm^2/sec, k = 10 sec^{-1} and initial particle density n_0 = 1 analog to a normalized intensity of 1, as function of distance from origin and time. One can clearly see the depletion zone for early times close to the bleached area in the simulated FLIP experiment, as well as the delayed onset of fluorescence loss distant from the bleached region. D, E, intensity profiles at selected distances from origin (grey symbols) with superimposed fit with the StrExp function (color coded for distance from 4 µm (red) to 11 µm (dark green) from origin, respectively).

We also calculated rate coefficients on a pixel-by pixel basis using self-programmed macros in ImageJ and the obtained parameter maps from the StrExp fit to data (i.e., *h*-map and *τ*-map). This involves calculating the exponent of one image to the other, which was coded in ImageJ macro language in a single loop as:

```
for (j=1; j<=200;j+=1) {
		s=0;
		w=getWidth();
		h=getHeight();
		for (y=0; y<h; y++) {
		for (x=0; x<w; x++) {
				v = getPixel(x, y);
				g=pow(v,p[s]);
				putPixel(x,y,g);
				s = s+1;
		}
		}
run("Next Slice [>]");
}
```

Here, p[s] is an array containing the exponent image (i.e., $\frac{1}{h}-1$) and s, and j are indices over the entries in that array and over the slice number, respectively. This operation was applied to a time stack (with the time resolution given by the respective experiment) divided by the *τ*-map. The resulting image of that operation was multiplied with the reciprocal of the product of the *h*-map and *τ*-map to get the final stack of rate coefficients. Since for the first image (t = 0) some values of the rate coefficients are infinite (see above), that frame was discarded before presentation (see below).

Bleach profile of the argon laser for varying objectives

We assessed the bleach/illumination profile of the laser in our confocal setup experimentally, using eGFP-expressing McA cells which had been fixed for 1h with 2% (w/v) paraformaldehyde at room temperature before imaging. A z-stack and bleach profile was subsequently acquired using either a 40x objective with 0.6NA or 1.2NA or a 63x objective with 1.2NA. For the bleaching, 20 iterations with 100% laser output over a circular area of 16x16 pixels for a pixel size of 0.1 µm giving a diameter of the circular bleach ROI of 1.6 µm were used (Fig. S2D, circle). After the bleach, we acquired a second z-stack, as shown in Fig. S2A-D. The results show clearly that for the low-NA objective,

the illumination profile is a cylinder, while it is a double cone for the high-NA objective. There is some broadening of the bleach profile in the fixed cells as indicated by the increasingly larger diameter of the bleached area compared to the size of the chosen bleach ROI (Fig. S2D, circles). That broadening could be due to residual slow diffusion of eGFP through the paraformaldehyde matrix. Alternatively, it could be due to scattering of excitation light during the intense bleach illumination. This is in fact supported by a recent systematic study of laser scattering during FRAP and FLIP experiments (3). The double cone shape of the bleach profile did not affect the fitting performance of the StrExp function to FLIP experiments, as verified for the 63x objective with NA = 1.2 (see Fig. 3 and 4) and for the 40x objective with NA = 1.2 (not shown but additional examples can be obtained from the authors upon request). Note, that we could not perform accurate diffusion-limited FLIP experiments for eGFP with the low NA objective, since the required faster scan speed would have further deteriorated the image quality for the low-NA objective.

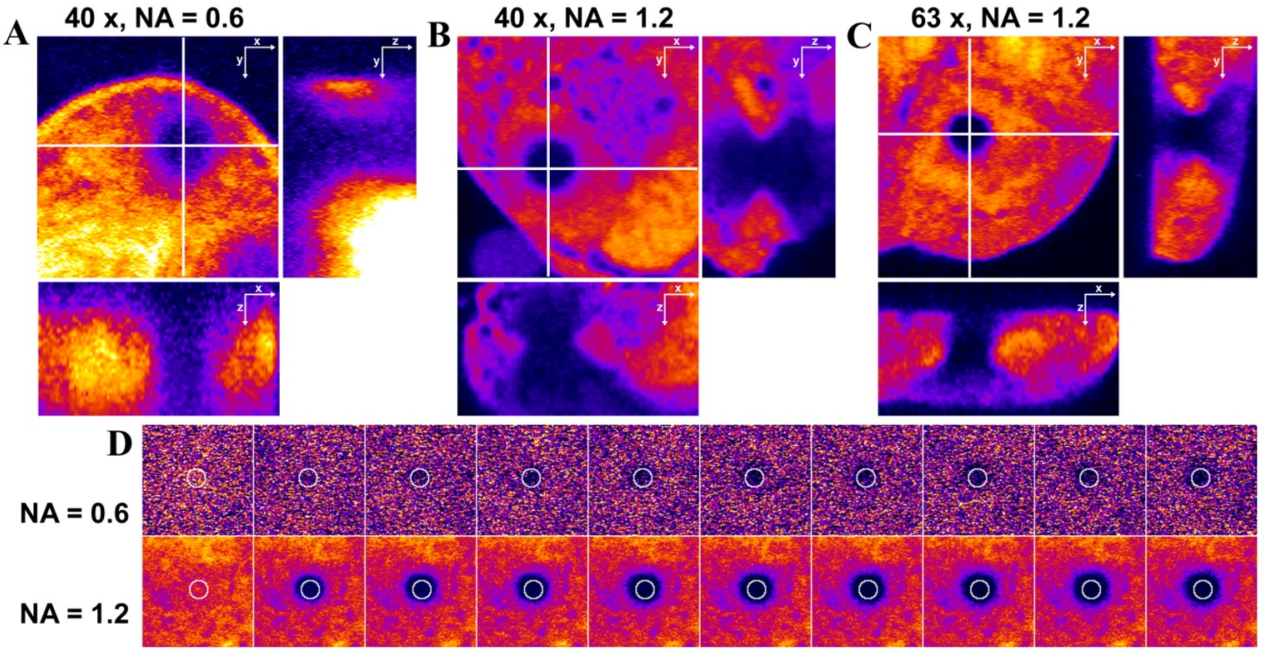

Fig. S2. Bleach profile of the Argon laser at 488 nm for various objectives.
McA cells expressing eGFP were fixed with2% (w/v) paraformaldehyde at room temperature before imaging at a confocal microscope with either a 40 x, NA = 0.6 (A, D, upper row), 40 x, NA = 1.2 (B, D, lower row) or a 63x, NA = 1.2 objective (C). Fluorescence of eGFP was bleached by 20 iterations with 100% output of the Argon laser over a circular area of 1.6 µm (white crosses in A-D). After bleaching, a z-stack consisting of 40 frames with z-step size of 0.5 µm was acquired for each objective. To eliminate bleaching during image recording, the laser power was reduced to 0.5 %. Two scans were averaged for each plane with a pixel dwell time of 1.6 µm during z-scanning for all objectives. D, montage of selected frames during the bleaching (shown is every 5th frame) reveals broadening of the bleach spot (white circle) during bleaching. This was most pronounced for the objective with higher NA (lower row in panel D). The size of the x,y-view images in A-C is 20x20 µm, while the (x,y),z-view images have dimension of 40x20 µm. The size of the images in panel D is 9.9x9.9 µm.

Three-dimensional simulation of diffusion-limited FLIP experiments

To assess the validity of the 2D approximation, we performed simulation of FLIP experiments in a 3D geometry. For that purpose, Eq. 7 of the main text was solved numerically in FENICS on a unit sphere with radius equal to 1 μm (Fig S3). As bleaching profile we chose either a cylinder with a bleach rate constant of k = 10 sec^{-1}, or we modelled a conical bleach profile, as observed for high NA objectives (Fig. S2B and C). The latter was approximated with a bleach rate constant exponentially decaying from the origin as $k = 20 \cdot \exp\left(-\sqrt{x^2 + y^2 + 4z^2}\right)$ sec^{-1}. The bleach radius was chosen to be $R = \sqrt{0.2 + a \cdot z^2}$ with $a = 0$ and $a = 0.2$ for the cylinder and cone shaped bleach profile, respectively. Outside that bleach radius, the bleach rate constant was zero. Diffusion constants were set as in the 2D simulation shown in Fig. 2 (i.e., D_1 = 0.2 μm^2/sec and D_2 = 0.8 μm^2/sec on the left and right half, respectively).

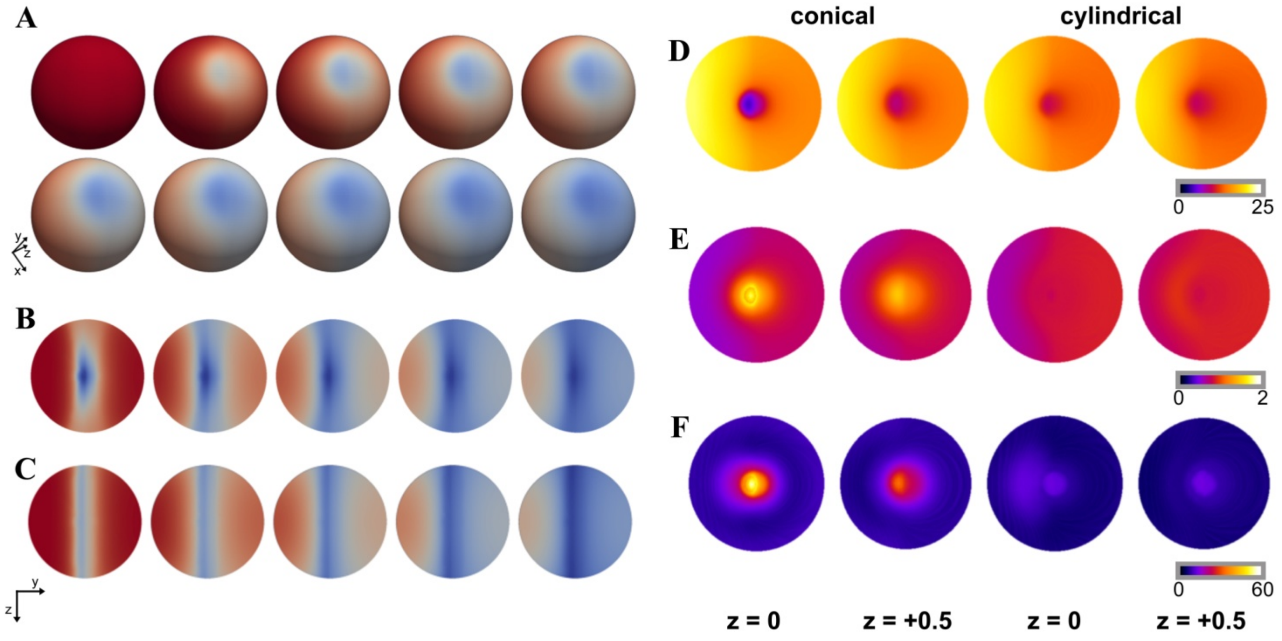

Fig. S3. 3D simulation of a FLIP experiment with heterogeneous diffusion in the unit sphere. A 3-dimensional bleaching experiment was simulated on a unit sphere with either a cylindrical or a conical bleach profile using FeniCS, an automated computational modelling suite (www.fenicsproject.org). The bleach radius was set to $R = \sqrt{0.2 + a \cdot z^2}$ with $a = 0$ and $a = 0.2$ for the cylinder and cone shaped bleach profile, respectively. The bleach rate constant and diffusion coefficients were set, as outlined in the text, below. A, 3D-view of the sphere showing selected snapshots for a FLIP simulation with the conical profile every 2 sec. Fluorescence loss is color-coded from red (high intensity) to blue (low intensity). B, C, selected snapshots taken every 5 sec parallel to the optical axis (i.e., y, z-sections through the unit sphere for the conical (B) and cylindrical bleach profile (C), respectively. D-F, the bleach-induced intensity decay was fitted for both bleach profiles with the StrExp function on a pixel-by-pixel basis using the PixBleach plugin to ImageJ. Two planes were selected fort he analysis; the equatorial plane (z = 0) and a plane above, at z = 0.5 μm, resembling the in-focus and an out-of-focus plane, respectively. The regression recovers a map of the time constant distribution color-coded between 0.0 to 25.0 sec (D), the stretching parameter, *h*-map, color-coded between 0.0 to 2.0 (E), and the χ^2-map color-coded between 0 to 60 (F). A FIRE-LUT was used for color-coding, where dark blue and yellow indicate lowest and highest values, respectively. Fitting results for the conical profile are shown on the two left panels and fort he cylindrical profile on the two right panels of (D-F), respectively. The equatorial plane has the larger diameter and is shown in the most left and second right panels of (D-F). The other panels (i.e. second left and most right in D-F) show fitting results for the plane at z = 0.5 μm. See text for further explanations.

Results of the 3D simulation

The reconstructed stack obtained from fitting the StrExp function to the two selected planes of the 3D simulation resembles exactly the simulated data set, and this was found for both bleaching profiles (not shown, but see the result for the corresponding 2D simulation in Fig. 2 of the main text). Time constant maps (Fig. S3D) and stretching *h*-maps (Fig. S3E) were overly comparable for both bleaching profiles. For both profiles, the time constants were larger for the left half of the sphere, where the diffusion constant is smaller. For that half and outside the bleach ROI, the recovered stretching parameters were smallest giving an overall strongly compressed exponential (i.e., a delayed) decay. All these results are in accordance with the 2D simulation shown in Fig. 2. A significant difference between the two bleach profiles is that inside the bleached region fluorescence decay was much faster for the conical than for cylindrical profile, and this difference was most pronounced for the equatorial (focal) plane (Fig. S3D; most left and second right panels). In that area, the fluorescence loss followed also the most stretched exponential decay (i.e., highest *h*-values) for the conical profile (Fig. S3E; most left and second right panels). The difference between both bleach profiles is less pronounced for the 'out-of-focus' plane at z = 0.5 μm. Fluorescence loss kinetics was much more compressed outside the bleach ROI in case of the conical compared to the cylindrical bleach profile (i.e. *h* ~ 0.5 and ~ 0.75 for the left and right half in the in-focus plane for conical bleach profile compared to i.e. *h* ~ 0.65 and ~ 0.85 for the left and right half in the in-focus plane for the cylindrical bleach profile). All these differences are a consequence of the higher bleach rate constant at the focal plane in the conical bleach profile mimicking the more focused laser beam of high NA objectives. For both bleach profiles outside the bleach ROI, fluorescence loss kinetics was slightly more compressed in the equatorial plane than in the out-of-focus plane. Together, the depletion zone is characterized by a gradual transition from stretched decay inside the bleach ROI to compressed decay outside the ROI. It is more pronounced for the left half with slower diffusion for both bleach profiles. For the conical bleach profile simulating FLIP experiments with high NA objectives, the depletion zone is more pronounced than for the cylindrical profile, and this is true for both halves (i.e., for both diffusion constants). The goodness of fit as judged by the Chi-Square values was also comparable with the exception of the bleached region for the conical profile, where the Chi-Square values were higher (see first two panels of Fig. S3F). In that region a biexponential fit gave better results (not shown) in support of the data in Fig. S1, above.

Diffusion-limited FLIP experiment of BODIPY-cholesterol in CHO cells

BODIPY-cholesterol (BChol) is a fluorescent analog of cholesterol, which resembles cholesterol in many respects including partitioning into physically different lipid phases in model membranes and intracellular trafficking (4). We showed recently that BChol, like the intrinsically fluorescent close cholesterol analog, dehydroergosterol (DHE) shuttles between surface and recycling endosomes by vesicular and non-vesicular transport (4). Two-photon time-lapse microscopy revealed heterogeneous diffusion of BChol in CHO cells with diffusion constants ranging from 0.01 μm^2/sec in proximity of the endocytic recycling compartment (ERC) to ~1.3 μm^2/sec in the plasma membrane (5). Thus, intracellular diffusion of BChol is several fold slower than that of eGFP. We performed a FLIP experiment of BChol in CHO cells, where the perinuclear vesicle pool resembling the ERC was repeatedly bleached and fluorescence loss monitored in the whole cell (Fig. S4). By fitting the StrExp function to that data several interesting aspects could be revealed:

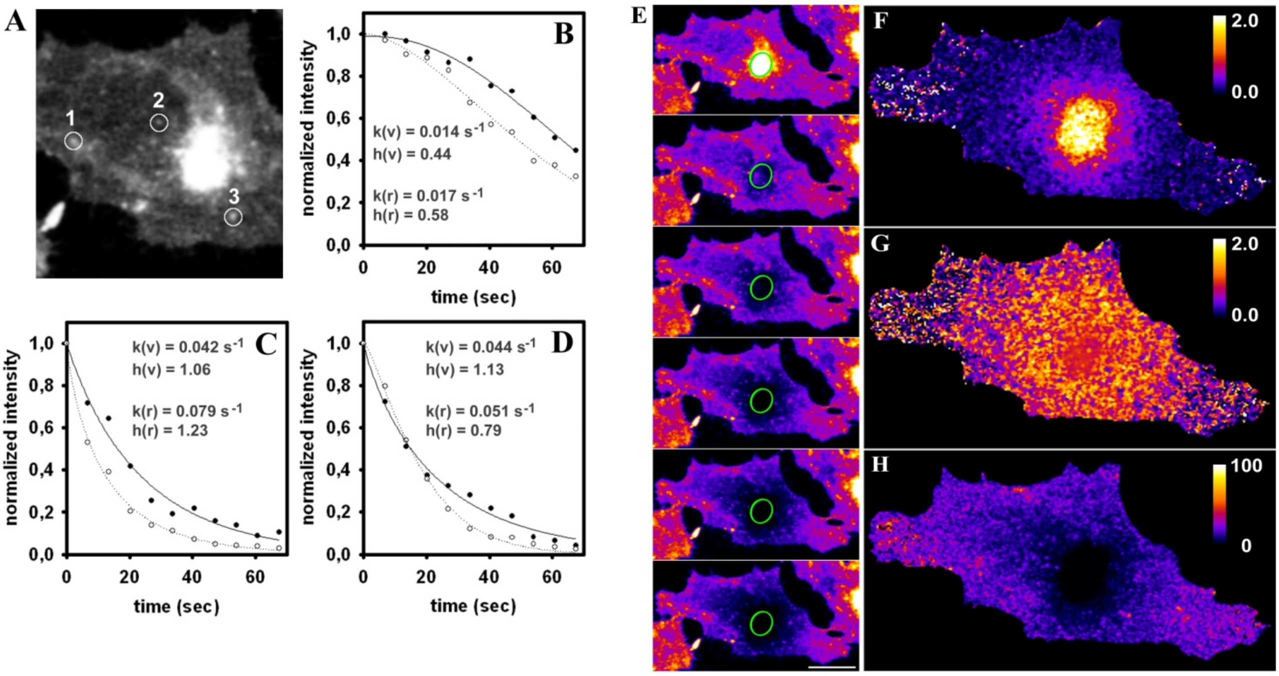

Fig. S4. FLIP experiment of BODIPY-cholesterol in CHO cells and fitting with the StrExp function.
Chinese hamster ovarian (CHO) cells were pulse-labeled with BChol using a sterol/cyclodextrin complex for 1 min at 37ºC, as described (3, 4). Cells were washed with buffer medium, chased for 30 min at 37ºC and placed at the stage of a confocal microscope. A FLIP experiment was performed as described for eGFP in McA cells (see Fig. 3) using the Argon laser line at 488 nm as excitation source. A-D, moving vesicles containing BChol were tracked manually or using SpotTracker as described in Materials and Methods. Intensity profiles of BChol in vesicles (‘v’) and a small region (5x5 pixels wide) surrounding the vesicles (‘r’) was measured and exported to SigmaPlot 9.0 (SPSS Inc, Chicago, IL, USA) for further analysis. In that program, the intensity profiles were fitted with the StrExp function giving rate constants, ‘k(v)’ and ‘k(r)’, and stretching parameters, ‘h(v)’ and ‘h(r)’, for the vesicles and the surrounding region, respectively. A, cell with tracked vesicles indicated with numbered circles for the respective region. The ERC is the bright vesicle pool between vesicle ‘2’ and ‘3’. B-D, fluorescence loss analysis for vesicles (closed symbols, data; straight line, StrExp fit) and surrounding region (open symbols, data; dotted line, StrExp fit) for region 1 (B), region 2 (C) and region 3 (D); compare panel A. E, First six frames of the FLIP image series color-coded using a FIRE LUT to reveal intensity changes (bleach ROI is indicated in green). Bar, 5 µm. F-H, selected results of pixel-wise fitting of the StrExp function using PixBleach. F, rate constant map (i..e., reciprocal of time constant map), G, *h*-map; H, map of RMSE values indicating goodness of fit. Values are color-coded using a FIRE-LUT, and the respective range is given by the scale bars.

1) fluorescence of BChol dropped first inside the bleach ROI, i.e., the ERC pool, and with some delay in the cytoplasm including cytoplasmic vesicles (Fig. S4 A-E). 2) Fluorescence loss in the cytoplasm and in vesicles could be well fitted with the StrExp function. Here, vesicles and cytoplasm far from the bleach ROI showed a compressed exponential intensity decay (see vesicle 1 in Fig. S4A, B). Closer to the bleached ERC, fluorescence loss of BChol could be well fitted with a stretched exponential decay, while intensity decay from the vesicles was slightly delayed compared to that in the cytoplasm (see vesicles 2 and 3 in Fig. S4A, C-D). These results suggest that sterol release from vesicles takes place during the FLIP experiment. Since the vesicles move, the quality of the pixel-wise fit with the StrExp function is low outside the bleach ROI (RMSE shown in Fig. S4H). Moreover, the ERC fluorescence was slightly saturated in the first frame, such that we could follow sterol also in tiny and dim cytoplasmic vesicles. This, however, affects the fitting outcome for the *h*-map inside the bleach ROI, where the values of the stretching parameter are too small. Nevertheless, the regression outcome clearly shows a gradient in bleach rate constants (reciprocal of time constant map, Fig. S4F). A gradual transition can be also found for the *h*-map with values larger than one close to the bleached region to values much lower than one towards the cell edge (Fig. S4G). Together, these results demonstrate that the StrExp function formalism accurately describes diffusion-limited FLIP experiments inside living cells.

Simulation of binding/barrier-limited FLIP experiments

The respective compartment model (Eq. 3-5 in Materials and Methods) was simulated using the following parameters: $k_1 = 0.025\,\text{sec}^{-1}; k_{-1} = 0.05\,\text{sec}^{-1}; k_2 = 0.25\,\text{sec}^{-1}$ (Fig. S5). The initial intensity in compartment 1 and 2 were $I_1^0 = 220$ and $I_2^0 = 110$, respectively, ensuring that $\frac{k_1}{k_{-1}} = \frac{I_2^0}{I_1^0}$ according to mass-action kinetics. Since in this FLIP simulation, diffusion is considered as being very fast (i.e., not rate-limiting), no depletion zone will be observed and fluorescence loss takes place homogeneously in the compartment where the bleach ROI is located. Accordingly, the ROI for the intended FLIP bleaching can be placed anywhere in compartment c_2. Gaussian noise was added to generate a realistic simulation on a pixel-by-pixel basis, and the synthetic FLIP data was fitted to a StrExp function for each pixel, as implemented in our ImageJ plugin ‘PixBleach’.

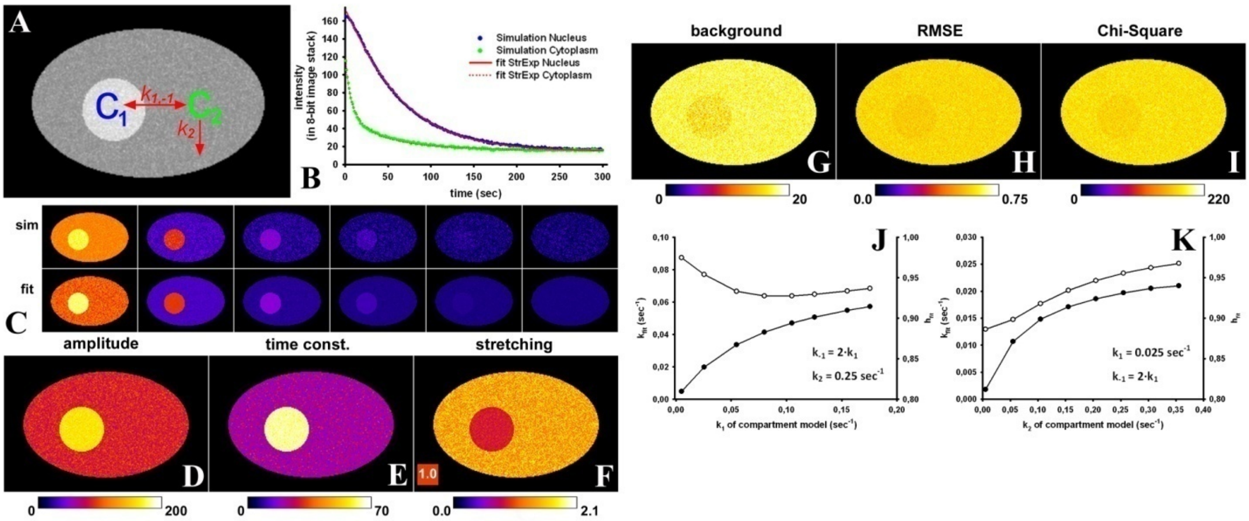

Fig. S5. Simulation of the homogeneous compartment model and fitting with the StrExp function.
A, The 2-compartment of Eq. 3-5 was simulated on a pixel-by-pixel basis using Macros in ImageJ with compartment 1 (‘C_1’) being separated from compartment 2 (‘C_2’), wherein a bleach region was arbitrarily placed. Bidirectional transport between both compartments occurs with rate constant k_1 and k_{-1}, respectively. Parameter values for the simulation were k_1 = 0.025 sec^{-1} and k_{-1} = 0.05 sec^{-1}, while bleaching in compartment 2 occurred with rate constant k_2 = 0.25 sec^{-1}. Initial values are given in the main text. Gaussian noise of standard deviation (SD) = 30 was added, and every frame represents simulated time points every second (i.e., frame 1 = 0 sec, frame 2 = 1 sec etc.). B, simulated intensity profile for a 10x10 box arbitrarily placed in compartment 1 (blue symbols) or compartment 2 (green symbols) were fitted with the StrExp function using SigmaPlot 9.0 (SPSS Inc, Chicago, IL, USA) for compartment 1 (blue line) or compartment 2 (green line). C, upper row shows the first 10 frames of the simulated image sequence (‘sim’), while the lower row shows the reconstructed data from fitting the StrExp function implemented in PixBleach to the data (‘fit’). D-E, parameter maps produced by PixBleach; D, amplitude map; E, time constant map; F, map of the stretching parameter (see Eq. 1; the color code for h = 1, the mono-exponential case, is given in small box)) in color-coding using a FIRE LUT for min-max values. For comparison, the color code for h = 1, the mono-exponential case, is given. G, H, map of the background values (G), RMSE values (H) and χ^2-values (I), the latter two showing the quality of the regression. All values are color-coded using a FIRE-LUT, and the range is given below the images with a color bar. The time constant is given in seconds; all other values are without units. J, K, rate constant (‘k_{fit}’; closed symbols) and stretching parameter (‘h_{fit}’; open symbols) as function of rate constants k_1 (J) and k_2 (K) of the compartment model given in Eq. 3-5. Other rate constants of the compartment model were set as given in the inset of panels J, K.

The reconstructed image stack using the estimated parameter sets resembled the synthetic FLIP data with high accuracy (Fig. S5B).The stretching factor, h, was slightly lower than 1 in compartment c_1 indicating a compressed exponential decay in this area (Fig. S5F). The recovered background image resembles the added Gaussian noise level and is homogenous (Fig. S5G). Root-mean-square-error (RMSE) and χ^2-map are also homogenous with low values for the given noise level indicating high fit quality (Fig. S5H, I). A systematic variation of the parameters for the compartment model followed by fitting with the StrExp function revealed the following: i) increasing the off-rate constant, k_1, increases the rate constant of the fit to the StrExp function in a non-linear manner (Fig. S5J); ii) increasing the bleach rate constant, k_2, compared to the off-rate constant, k_1, also increases the rate constant of the fit to the StrExp function in a non-linear manner (Fig. S5J); iii) compressed exponential decays (i.e., with fitted stretching parameter, h < 1.0) are

most pronounced, if $k_2 \sim k_1$ or even $k_2 < k_1$. Additional simulations showed that increasing the backward rate constant, k_{-1}, for constant values of k_1 and k_2 only affects the amplitude parameter of the fit to the StrExp function (not shown).

To assess the fitting performance of the StrExp function to binding/barrier-limited FLIP experiments, we systematically varied the noise level in the simulated time series.   .

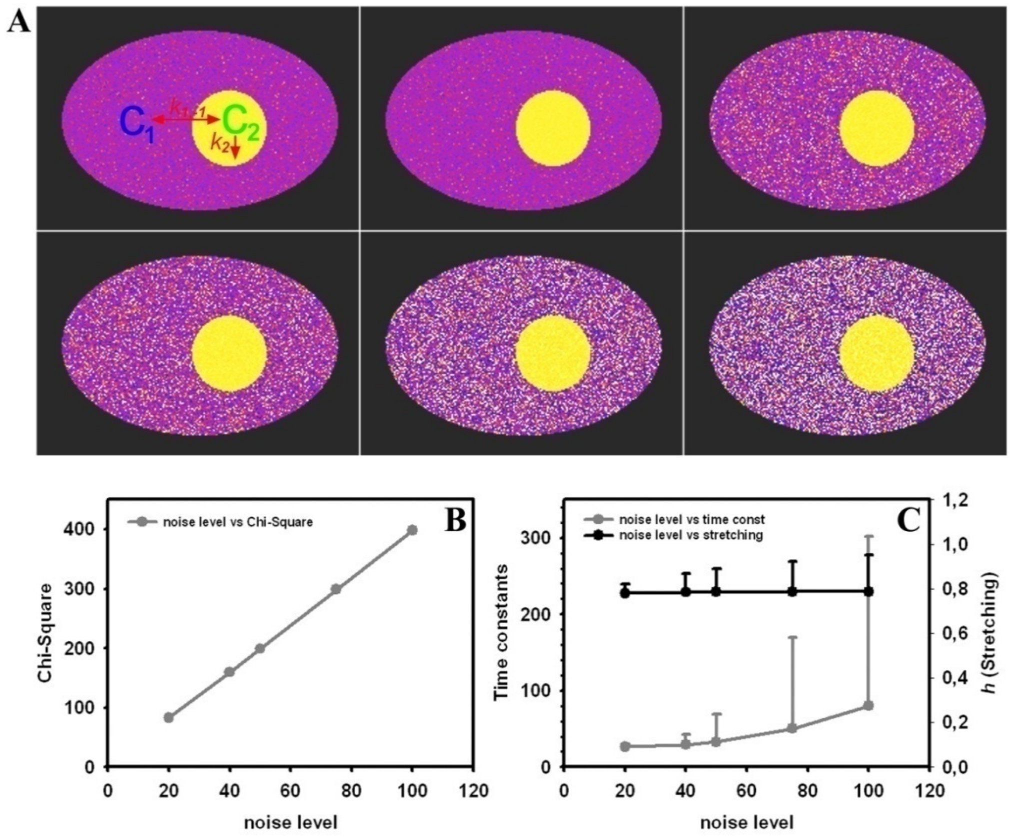

Fig. S6. Effect of additive image noise on fitting performance. A compartment model similar to that in Fig. S5 was simulated with additive Gaussian noise of increasing variance. A, simulation output for the first frame with SD = 20 (upper most left and middle panel), 30 (upper right panel), 50, 75 and 100 (lower panels from left to right). B, Chi-Square of the StrExp fit to the simulated image sets for a ROI placed in C_1. C, estimated time constant and stretching values, as recovered from the StrExp fit to the same region in C_1.

First, Gaussian noise of increasing variance was added to the image stacks as first approximation for detector noise, and the simulated data sets were subsequently fitted with the StrExp function (Fig. S6). Clearly, the fit is reliable for noise levels up to SD = 50, above which the time constants were overestimated and associated with large uncertainty, as indicated by the high standard deviation (grey curve in Fig. S6C). Estimation of the stretching parameter was slightly more robust against additive Gaussian image noise (black curve in Fig. S6C).

Noise can be defined in a broad sense as random fluctuation on a signal in space and/or time. To account for locally heterogeneous time courses of fluorescence loss, we implemented next the compartment model on a pixel-by-pixel basis and varied the initial concentrations at each location stochastically (Fig. S7). That was done by adding a value, g, multiplied by a random number drawn from Gaussian distribution with zero mean and unit variance to the initial concentration at each pixel position.

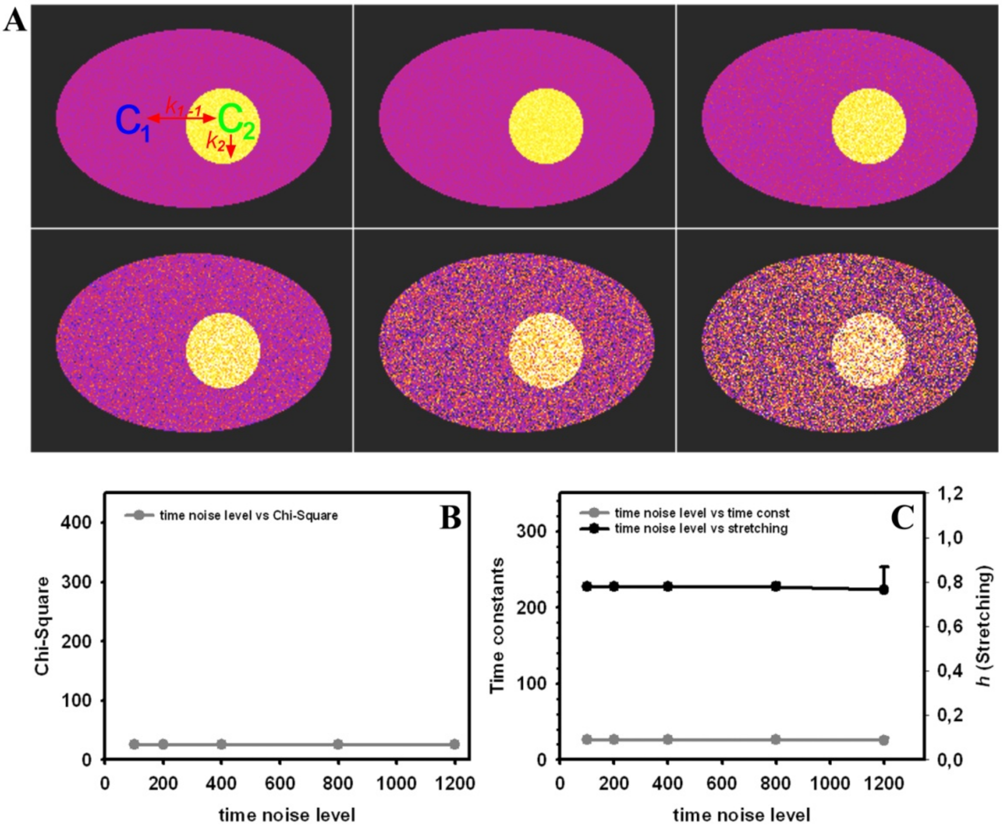

Fig. S7. Effect of time noise on fitting performance. The model of Fig. S6 was simulated with different parameters at every pixel position and fitted with the StrExp function, as explained in the text. The initial intensity in C_1 and C_2 of 2600 and 3800, respectively, were varied at each pixel position by adding a number g = 100 to 1200 multiplied with a random number chosen from a Gaussian distribution with zero mean and unit variance. This also varied $k_{-1}=C_1^0/C_2^0$, which is why we call this approach ‚time noise‘.

B, Chi-Square of the StrExp fit to the simulated image sets for a ROI placed in C_1. C, estimated time constant and stretching parameters, as recovered from the StrExp fit to the same region in C_1.

Due to the mass-action principle, varying the initial fluorophore amount also varied the rate constant k_{-1} from pixel to pixel. That is, we have created ‘noise’ by pixel-to-pixel variation in the initial concentrations $C_1^0 \rightarrow C_1^0(x,y)$ and $C_2^0 \rightarrow C_2^0(x,y)$ which, thus consist of a deterministic and a stochastic part. We get spatially varying time courses due to the relation

$$k_{-1} \rightarrow k_{-1}(x,y) = {k_1 \cdot C_1^0(x,y)} \Big/ {C_2^0(x,y)}. \qquad \text{(S5)}$$

Accordingly, every pixel experienced different fluorescence loss kinetics. The forward rate constant, k_1, was kept fixed at k_1 = 0.025sec^{-1}. Every call of the program generates another parameter combination for each pixel giving different time courses and image stacks. The relation between all pixels in the respective region, however (i.e. inside and outside the bleach ROI), remains deterministic. We call that model for ‘compartment model with time noise’ (see Fig. S7). When fitting the StrExp function to this synthetic FLIP data, we found very good agreement (i.e., the Chi-square values were in the same range as for additive Gaussian noise in the homogeneous compartment model with SD =10; compare Fig. S6B and S7B). When raising the stochastic contribution to the initial concentrations

and thereby the heterogeneity of time courses due to increasing pixel-to-pixel variation of k_{-1}, the amplitude maps (Fig. S8A-F) and time constants maps (Fig. S8A'-F') were increasingly structured. In addition, amplitude and time constant maps were positively correlated (i.e., we recovered image-based Pearson correlation coefficients of 0.85 – 0.97). This resembles the findings for experimental FLIP image sets of eGFP (see Fig. 4 and Fig. S9). The *h*-map, however, remained in a narrow range of $h \sim 0.75$-0.77 and $h \sim 1.0$ for all pixels in compartment 1 and 2, respectively. This is in accordance with the homogeneous compartment model, but it is in disagreement with the experimental observations for eGFP in cells (compare Fig. 4 and 5 of the main text).

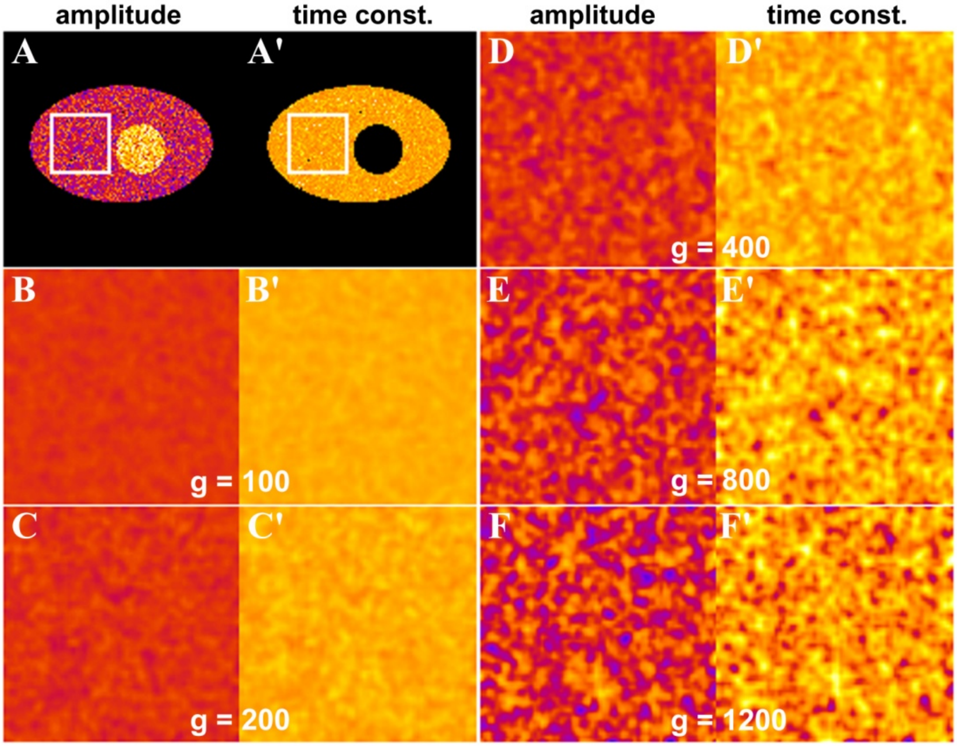

Fig. S8. Correlation between amplitude and time constant maps in the StrExp fit to the spatially heterogeneous model. The compartment model of Fig. S7 was simulated with increasing variation of the initial intensity in compartment 1 and 2 followed by fitting with the StrExp function as specified in legend to Fig. S6B, C. From the StrExp fit, the amplitude (A-F) and time constant maps converted to an 8-bit FIRE LUT (A'-F') and contrast-adjusted to reveal the similarity in structures are shown. B-F' show the boxes in A, A' enlarged.

Detection of diffusion barriers for eGFP in the nucleus of McA cells by FLIP analysis

By performing binding/barrier-limited FLIP experiments in the nucleus of eGFP expressing McA cells, we found that regions with high nuclear eGFP content experience slower fluorescence loss (Fig. S9). This was found irrespective of the location of the bleach spot (i.e., whether it was placed in regions of the nucleus with low eGFP content (Fig. S9A-G) or with high eGFP content (Fig. S9H-L'). Regions with intermediate to low eGFP content are rich in DNA, as revealed by co-staining with Hoechst33342, a live-cell DNA stain (Fig. S9H, eGFP in green and Hoechst33342 in red). By fitting the StrExp function pixel-wise to the data set, we found a positive correlation of amplitude and time constant map (Fig. S9I). Very low amplitude values likely correspond to the nucleoli, where the correlation is low.

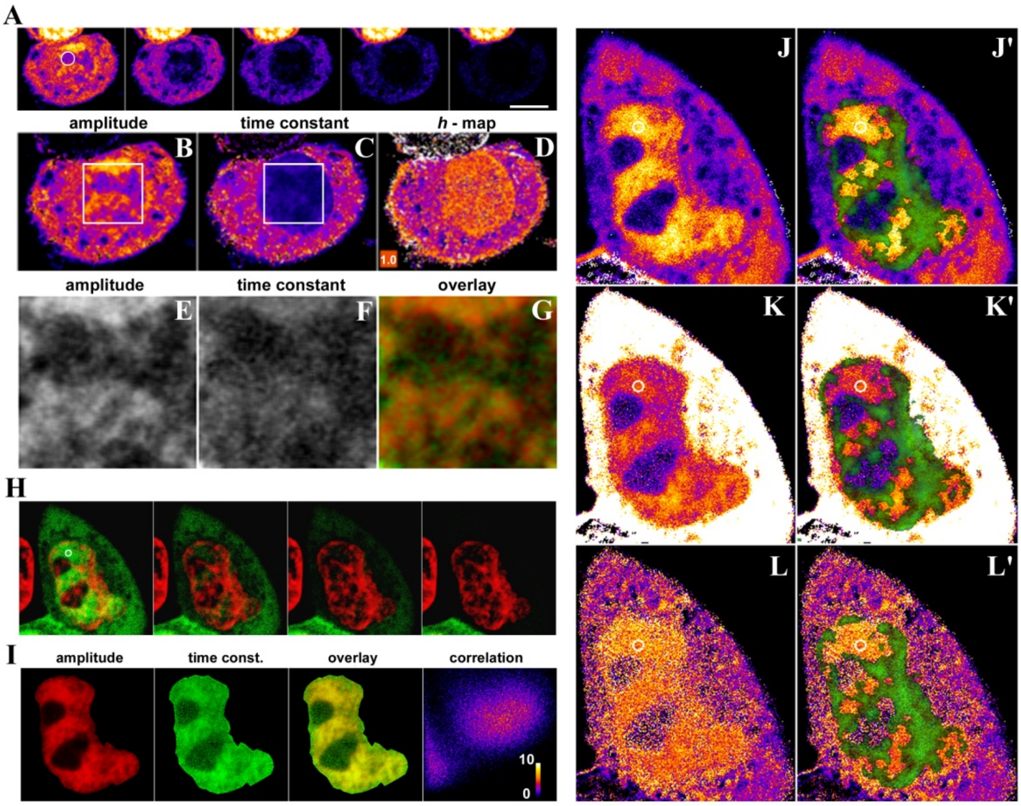

Fig. S9. Pixel-wise FLIP analysis of eGFP in the nucleus. McArdle RH7777 cells expressing eGFP in the cytoplasm and nucleus were placed on a temperature-controlled stage of a confocal microscope maintained at 35 ± 1ºC. A circular region of 30 pixel diameter in the nucleus (white circle) was repeatedly bleached with full laser power. The total frame rate was 3.2 sec. A montage of every 20th frame of the data. The data was fitted pixel-wise to a StrExp function. B, amplitude map; C, time constant map; D, map of the stretching parameter (see Eq. 1; the color code for *h* = 1, the mono-exponential case, is given in small box). All values are color-coded using a FIRE-LUT. The range of the time constant map is between 0 sec to 150 sec. E-G, zoom of amplitude map (E) and time constant map (F) and color overlay with amplitude in red and time constant in green for rectangular box in panels B-D. Bar, 10 µm. H-L'. a separate experiment with similar settings where cells were co-stained with Hoechst 33342 to visualize the DNA. While eGFP was imaged and bleached with the 488-nm line of an Argon laser, Hoechst 33342 was excited by a two-photon process with 720 nm from a pulsed Ti:Sapphire laser (MaiTai, Spectra Physics). H, montage of every 20th frame of the data with eGFP in green and Hoechst 33342 in red. I-L', comparison of parameter maps obtained by pixel-wise fitting of the StrExp function to fluorescence loss of eGFP with focus on nuclear fluorescence. I, correlation analysis of nuclear amplitude and time constant map. The most right panel shows a scatter plot of 8-bit converted values for the amplitude (abscissa) and time constant (ordinate) with coincidence binned in a range of 0-10 and color-coded using a FIRE LUT. The pearson coefficient for that region was determined to r = 0.875. J, J' amplitude; K,K' time constant and L, L' stretching parameters for the same experiment in RGB format with FIRE LUT. On the respective right panels (i.e., J', K', L') the segmented Hoechst image was overlaid (in green) to visualize that regions with high DNA content have proportionally low eGFP content and experience a more rapid fluorescence loss. Note that the time constant maps were scaled from $\tau = 0$ sec to $\tau = 5$ sec for visualization purposes. Therefore, the cytoplasm appeared saturated, since in the cytoplasm fluorescence loss was much slower (i.e., $\tau \sim 10$ sec).

Supporting references

1. Lee, K. C., Siegel, J., Webb, S. E., Lévêque-Fort, S., Cole, M. J., Jones, R., Dowling, K., Lever, M. J., and French, P. M. (2001) Application of the stretched exponential function to fluorescence lifetime imaging., *Biophys. J. 81*, 1265-1274.

2. Berberan-Santos, M. N., Bodunov, E. N., and Valeur, B. (2005) Mathematical functions for the analysis of luminescence decays with underlying distributions 1. Kohlrausch decay function (stretched exponential). *Chemical Physics 315*, 171-182.

3. Dobrucki, J. W., D. Feret, and A. Noatynska. 2007. Scattering of exciting light by live cells in fluorescence confocal imaging: phototoxic effects and relevance for FRAP studies. Biophys. J. 93:1778-1786.

4. Wüstner, D., Solanko, L., Sokol, E., Garvik, O., Li, Z, Bittman, R., Korte, T. and Herrmann, A. 2011. Quantitative Assessment of Sterol Traffic in Living Cells by Dual Labeling with Dehydroergosterol and BODIPY-cholesterol. *Chem. Phys. Lipids. 164, 221-235.*

5. Lund, F.W., Lomholt, M.A., Solanko, L. M., Bittman, R. and Wüstner, D. 2012. Two-Photon Time-Lapse Microscopy of BODIPY-Cholesterol Reveals Anomalous Sterol Diffusion in Chinese Hamster Ovary Cells. *BMC Biophysics*, *In press.*

Movie 1.

Numerical simulation of FLIP experiment with space-dependent diffusion coefficient

A 2-dimensional bleaching experiment was simulated on a disk with a circular bleached area of radius $r_1 = 0.5$ µm using FeniCS, an automated computational modelling suite (www.fenicsproject.org). The bleaching rate is set to $k = 10$ sec^{-1} and the diffusion coefficient is $d_1 = 0.2$ µm^2/sec and $d_2 = 0.8$ µm^2/sec on the left and right half disk, respectively. The simulation is shown in the left panel starting after the first bleach and intensity color-coded with a FIRE-LUT. The right panel shows the time evolution of the intensity profile along a horizontal line through the equator of the 2D disk. The movie is played with 1 frame per sec.

Movie 2.

Time course of barrier-limited FLIP experiment of eGFP

McArdle RH7777 cells expressing eGFP in the cytoplasm and nucleus were placed on a temperature-controlled stage of a confocal microscope maintained at 35 ± 1ºC. A 30 pixel diameter circular region in the cytoplasm (white circle) was repeatedly bleached with full laser power, while the whole field was scanned with 0.5% laser output between the bleach scans such that the total frame rate was 2.6 sec. The movie is played with 15 frames per sec.

Movie 3.

Time evolution of rate coefficients for barrier-limited FLIP experiment of eGFP

From the pixel-wise fitting of the StrExp function to FLIP of eGFP in McA cells (Fig. 4 of main text), the rate coefficients were calculated on a pixel-by-pixel basis using Eq. S4, above and self-programmed macros to ImageJ. Rate coefficients are color-coded using a FIRE-LUT. Accelerating fluorescence loss kinetics in some subcellular regions can be inferred from a color shift from blue (low speed) to yellow and white (high speed) over time. The movie is played with 15 frames per sec.
